# Neutrophil Extracellular Traps in Atherosclerosis and Cardiovascular Disease

**DOI:** 10.1002/mco2.70912

**Published:** 2026-09-15

**Authors:** Ji Zhang, Fangjiaqi Wei, Mengdi Qu, Yuwen Shao, Tingye Xu, Darko Stojkov, Hans‐Uwe Simon, Ben Huang, Changhong Miao, Hao Zhang

**Affiliations:** ^1^ Department of Anesthesiology Zhongshan Hospital Fudan University Shanghai China; ^2^ Shanghai Key Laboratory of Perioperative Stress and Protection Shanghai China; ^3^ Department of Anesthesiology Shanghai Medical College Fudan University Shanghai China; ^4^ Institute of Pharmacology University of Bern Bern Switzerland; ^5^ Institute of Biochemistry Brandenburg Medical School Neuruppin Germany; ^6^ Department of Cardiovascular Zhongshan Hospital Fudan University Shanghai China

**Keywords:** atherosclerosis, cardiovascular disease, immunothrombosis, neutrophil extracellular traps, precision therapy

## Abstract

Atherosclerosis is a chronic inflammatory disease of the arterial wall and the leading pathological basis of cardiovascular morbidity and mortality worldwide. Neutrophil extracellular traps (NETs), web‐like chromatin structures armed with histones, myeloperoxidase, and serine proteases, have emerged as active participants in vascular inflammation and thrombosis. Under physiological conditions, NETs contribute to host defense and immunothrombosis, but excessive or persistent NET formation drives endothelial injury, plaque progression, and acute coronary events. However, how NETs shift from protective to pathological across the atherosclerotic continuum, and how their individual components cooperate to amplify vascular damage, remain insufficiently understood. This review explores NET homeostasis in the cardiovascular system and the balance between protective and pathological NET release. We then describe the molecular mechanisms of NET formation and the cooperative actions of NET components as an integrated thrombo‐inflammatory platform. Next, we discuss stage‐specific NET roles throughout atherogenesis, plaque destabilization, and ischemia–reperfusion injury. Finally, we evaluate clinical and translational evidence linking NET biomarkers to cardiovascular outcomes and critically assess precision‐targeted NET therapies. This framework highlights NETs as both mechanistic drivers and promising therapeutic targets in atherosclerotic cardiovascular disease.

## Graphical abstract

NETs as integrated effectors in the atherosclerotic cardiovascular disease continuum. Activated neutrophils release NETs that promote endothelial injury, thrombo‐inflammation, and plaque destabilization throughout atherosclerotic disease progression. Created with BioRender.com.

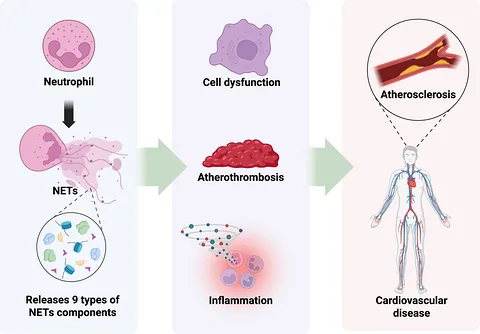

## 1 Introduction

Atherosclerosis (AS) remains the pathological basis of most ischemic cardiovascular events and continues to impose a major global health burden [1, 2]. It is now recognized not as a passive disorder of lipid deposition, but as a chronic inflammatory disease of medium and large sized arteries in which dysregulated lipid handling, endothelial injury, immune activation, fibrous remodeling, and calcification evolve in parallel [3, 4]. As plaques progress, they become increasingly prone to erosion, rupture, and thrombosis, thereby driving myocardial infarction(MI), stroke, and other major adverse cardiovascular events [5, 6].

Neutrophil extracellular traps (NETs) have emerged as an important link between innate immunity and vascular injury in AS [7, 8]. NETs are extracellular web‐like structures composed of decondensed chromatin decorated with histones and neutrophil‐derived effector proteins [7]. Their formation can be triggered through several pathways, among which reactive oxygen species (ROS) generation and peptidylarginine deiminase 4 (PAD4)‐dependent chromatin decondensation remain central mechanisms [9]. NETs are involved in pathological processes associated with various diseases, such as autoimmune diseases [10], chronic inflammatory diseases [11, 12, 13], diabetes [14], Alzheimer's disease [15], and cancer [16]. In the atherosclerotic milieu, NETs do more than mark neutrophil activation [17]. They amplify sterile inflammation, injure endothelial cells, activate macrophage inflammatory programs, promote plaque progression, and foster immunothrombosis. Recent reviews also support a role for NETs in shaping a plaque microenvironment that favors lipid accumulation and foam cell formation, although the contribution of individual NET‐associated components appears to be context dependent [18, 19].

Although research on NETs in the cardiovascular system has expanded considerably, many aspects of their biology remain incompletely understood. Their effects are unlikely to be uniform across disease stages, and the relative contribution of the intact NET scaffold versus individual NET‐associated mediators still requires clearer definition. In this review, we examine NET biology across the continuum of atherosclerotic cardiovascular disease (CVD), from vascular homeostasis to plaque progression, thrombosis, and postinfarction remodeling. Rather than discussing NET components as isolated entities, we emphasize their cooperative functions, disease‐stage specificity, and translational implications. This framework aims to clarify what makes NETs a distinct driver of cardiovascular pathology and where their biology may be therapeutically actionable.

## 2 NETs in Cardiovascular Homeostasis

Before examining how dysregulated NET formation contributes to vascular disease, it is essential to understand the role of NETs under physiological conditions. This section outlines the homeostatic functions of NETs in the cardiovascular system. We first describe their protective roles in host defense and vascular surveillance (Section 2.1), then define the thresholds that separate beneficial from pathological NET formation (Section 2.2), summarize the regulatory mechanisms that maintain NET homeostasis (Section 2.3), and finally consider how this balance shapes cardiovascular immune equilibrium (Section 2.4). Framing NETs first as physiological effectors provides the basis for understanding their later transition toward a pathological phenotype in AS.

### 2.1 Physiological Roles in Host Defense and Vascular Surveillance

NETs were first identified as a defense mechanism against invading pathogens [20]. Under physiological conditions, their formation is tightly regulated. NETs immobilize and neutralize bacteria, fungi [21], protozoa [22], and viruses [23] through the antimicrobial proteins embedded within their chromatin scaffold. Rather than killing pathogens directly in every case, NETs concentrate these effectors at infection sites and create a localized killing zone that works alongside phagocytosis to prevent systemic spread [24, 25]. Moreover, neutrophils are now recognized as active participants in immune surveillance and tissue homeostasis. Homeostatic neutrophil populations have been identified in uninfected lymph nodes [10], and neutrophil–endothelial interactions are central to leukocyte recruitment during sterile inflammation and vascular stress [11]. Recent evidence shows that dietary fluctuations can reprogram bone marrow granulopoiesis and alter neutrophil phenotypes in ways that directly influence vascular inflammation [12, 13]. These observations suggest that tightly regulated neutrophil activation may contribute to vascular immune surveillance [14], though whether this reflects a dedicated homeostatic program or an early response to subclinical injury has not been directly established. The close relationship between innate immune activation and hemostatic regulation further implies that neutrophil responses may influence endovascular integrity [15, 16], but the specific contribution of NETs under nonpathological conditions remains incompletely understood.

### 2.2 Thresholds Between Protective and Pathological NET Formation

**FIGURE 1:**
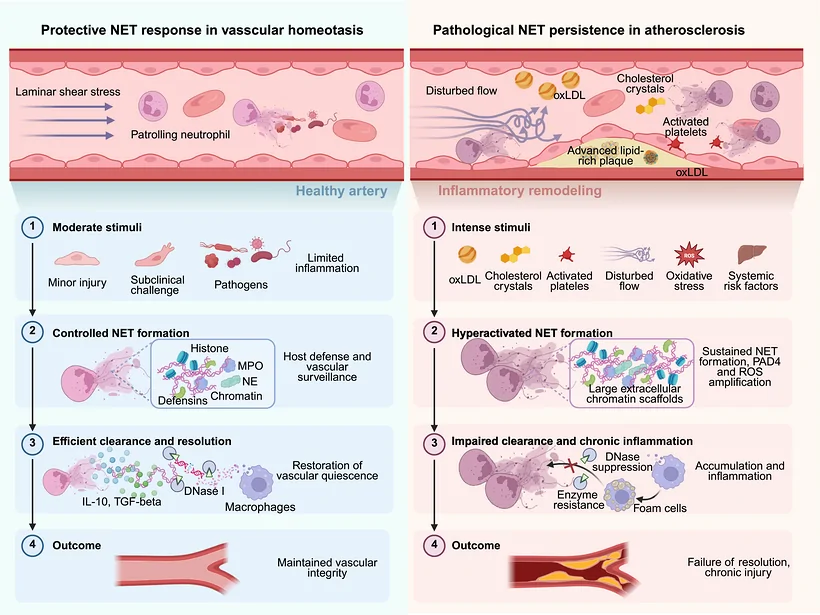
Protective‐to‐pathological transition of NETs in vascular homeostasis and atherosclerosis. Under physiological conditions, DNase‐mediated degradation and macrophage phagocytosis resolve NETs and maintain vascular homeostasis. In atherosclerosis, oxLDL, cholesterol crystals, activated platelets, and disturbed flow promote sustained NET formation, while impaired clearance by foam cells allows NET persistence, shifting NETs from a transient defense response to a chronic driver of vascular inflammation. *Created with BioRender.com. DNase, deoxyribonuclease; oxLDL, oxidized low‐density lipoprotein; NET, neutrophil extracellular trap; PAD4, peptidylarginine deiminase 4; ROS, reactive oxygen species.

Whether NET formation confers protection or drives tissue injury depends on the balance between induction strength and clearance efficiency (Figure 1). Under normal conditions, moderate stimulation triggers a controlled NET response that resolves once the danger signal is eliminated through enzymatic degradation and phagocytic clearance [17, 26, 27]. This balance breaks down in AS. The arterial wall in atherosclerotic disease contains multiple stimuli that push neutrophils toward sustained NET formation. Oxidized low‐density lipoprotein (oxLDL) and related oxidized phospholipids can promote NET release, while cholesterol crystals provide an additional trigger that links lipid accumulation to neutrophil activation [28, 29]. Activated platelets further amplify this response through platelet–neutrophil crosstalk [30]. Disturbed blood flow at arterial branch points amplifies endothelial activation and neutrophil recruitment [31]. Cardiometabolic risk factors lower this threshold even further. Diabetes primes neutrophils for exaggerated NET formation [32], hypercholesterolemia impairs NET clearance [33], and tobacco exposure has also been linked to enhanced NET formation [34]. As a result, the problem in AS is not only increased NET generation, but also prolonged NET residence within the vascular compartment.

At the same time, the capacity to clear NET remnants declines through multiple converging mechanisms [33, 35, 36]. The outcome is not simply more NETs, but NETs that persist in a state mechanistically distinct from the transient physiological response. Several molecular factors set this threshold. The two principal intracellular drivers of NET formation, PAD4‐mediated histone citrullination and ROS generation, are both elevated under diabetic and hyperlipidemic conditions [37]. ROS production scales with stimulus strength, amplifying NET release in proportion to the inflammatory insult [38]. Together, these factors create an individual inflammatory set point that varies with metabolic status, which may help explain the wide interpatient variability in NET‐associated cardiovascular outcomes.

### 2.3 Regulatory Mechanisms Maintaining NET Homeostasis

Several mechanisms work in parallel to resolve NET formation and prevent the buildup of immunostimulatory debris. Circulating DNase I degrades the DNA backbone of NETs, breaking down the chromatin scaffold and limiting the availability of NET‐associated danger signals [17, 30, 39]. DNASE1L3, produced by macrophages and dendritic cells, handles protein‐bound DNA that DNase I cannot efficiently process. DNASE1L3 deficiency is linked to autoimmune disease, and recent studies have also connected it to features of accelerated vascular inflammation [40, 41, 42, 43]. Within atherosclerotic plaques, the oxidative and protease‐rich environment may impair enzymatic degradation of NET components, allowing transient scaffolds to become more persistent. A study showed that NET protein components can adhere to vascular endothelium for months after a single inflammatory insult, while detached extracellular DNA continues to circulate over even longer periods [44]. These findings underscore the difficulty of clearing NET remnants from the vascular compartment once they form.

Macrophages provide a parallel line of defense by engulfing NET remnants through scavenger receptor‐dependent phagocytosis. This capacity is compromised in the lipid‐loaded foam cells that populate atherosclerotic lesions [33, 45]. Anti‐inflammatory cytokines such as interleukin‐10 (IL‐10) and transforming growth factor‐β (TGF‐β) normally suppress neutrophil activation during the resolution phase, but these pathways are blunted by the persistent inflammatory environment of advanced plaques [46]. Endothelial signaling also contributes to restraining neutrophil responses under resting conditions, though which molecular checkpoints are involved and how they fail during atherogenesis requires further study [47]. Regulatory T cells have been proposed to suppress NETosis through immunosuppressive mediators [48], but their specific role in the atherosclerotic vascular bed remains to be established.

When these mechanisms fail together as they do in advanced AS, the result is a vascular environment in which NETs accumulate rather than resolve, shifting from a transient immune response to a sustained driver of inflammation.

### 2.4 Implications for Cardiovascular Immune Balance

The preceding sections establish that the pathogenic impact of NETs in AS arises from the mismatch between sustained formation and impaired clearance, rather than from overproduction alone. This imbalance may not be merely a consequence of established disease. Notably, contrary to earlier models depicting low NET abundance in initial lesions, recent evidence indicates that NETs are abundant during early atherogenesis and may precede the transition to macrophage‐dominated extracellular traps in more advanced lesions [29, 49, 50]. Early defects in NET clearance and chronic neutrophil priming could therefore act as permissive contributors during subclinical atherogenesis, amplifying low‐grade vascular inflammation before overt plaque formation becomes morphologically evident [51, 52]. Thus, restoring NET homeostasis, rather than simply blocking NET formation may represent a more physiologically appropriate therapeutic target.

## 3 Molecular Mechanisms of NET Formation and Regulation

**FIGURE 2:**
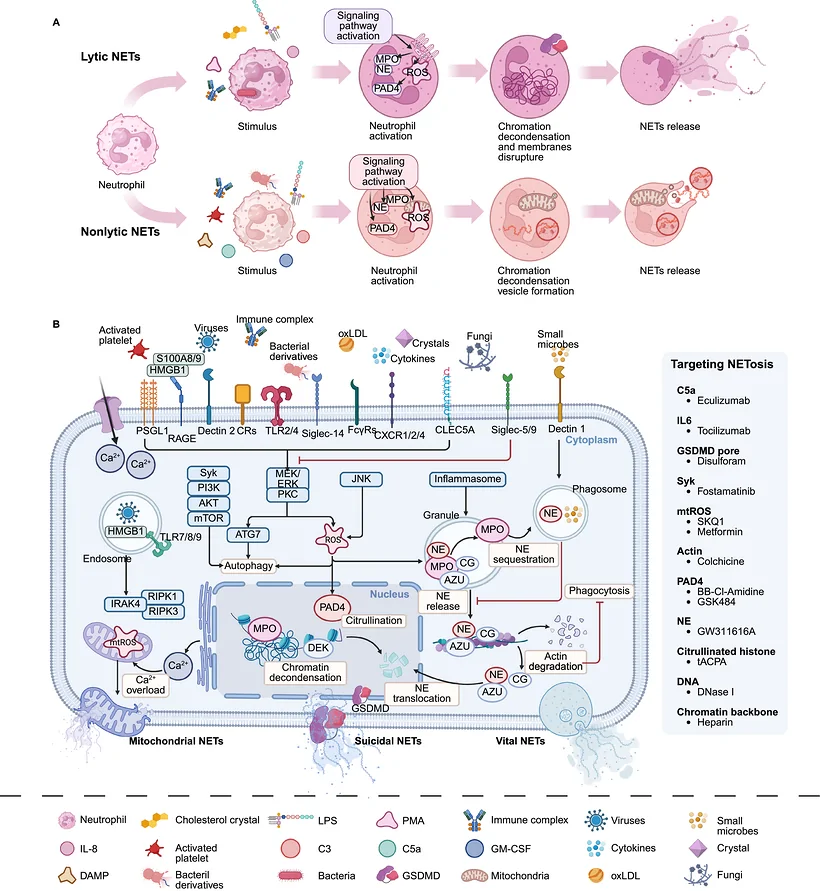
Mechanisms and regulatory pathways of NET formation. (A) Lytic and nonlytic pathways of NET release. In the lytic pathway, PAD4‐mediated chromatin decondensation leads to membrane rupture and neutrophil cytolysis. In the nonlytic pathway, chromatin or mitochondrial DNA is extruded via vesicles, preserving cell viability. (B) Integrated signaling networks driving NET formation. Diverse stimuli engage surface receptors and activate downstream kinases, converging on ROS production, calcium flux, PAD4‐mediated histone citrullination, and chromatin decondensation, ultimately leading to NET formation. Key pharmacological targets are indicated. *Created with BioRender.com. DAMP, damage‐associated molecular pattern; GSDMD, gasdermin D; MPO, myeloperoxidase; mROS, mitochondrial ROS; NE, neutrophil elastase; PAD4, peptidylarginine deiminase 4; ROS, reactive oxygen species; TLR, Toll‐like receptor.

Collectively, NET formation can be understood as a multilayered process that integrates metabolic priming, ROS‐dependent signaling, chromatin remodeling, and regulated membrane rupture. These events form a hierarchical cascade rather than independent modules, and the specific stimulus, metabolic state, and tissue context determine the extent and mode of NET release. Recent studies have clarified the metabolic requirements, epigenetic regulators, and cell death pathways that shape this process, with growing relevance to thrombo‐inflammatory diseases such as AS. In this section, we discuss the cellular and metabolic drivers of NET formation, the role of PAD4 in chromatin remodeling, and the emerging crosstalk between NET formation and other regulated cell death pathways (Figure 2B).

### 3.1 Cellular and Metabolic Drivers of NET Formation

As described above, NET formation can proceed through lytic (suicidal) or vital (nonlytic) pathways, each with distinct signaling requirements (Figure 2). Suicidal NETosis depends heavily on nicotinamide adenine dinucleotide phosphate (NADPH) oxidase‐generated ROS, including superoxide and hydrogen peroxide [53, 54]. Neutrophils from patients with chronic granulomatous disease, who carry mutations that impair NADPH oxidase function, fail to undergo this form of NET release [54]. In contrast, when neutrophils are stimulated through NADPH oxidase‐independent routes, mitochondria‐derived ROS become the major oxidative source for NET production [55, 56]. Vital NET formation occurs more rapidly, typically within 5–60 min, and preserves membrane integrity and cell viability [57, 58]. A mechanistically distinct nonlytic pathway, first identified in 2009 by Yousefi et al., involves the selective externalization of mitochondrial DNA (mtDNA) rather than nuclear chromatin, in an ROS‐dependent manner [59, 60]. Although both processes are nonlytic, mtDNA‐based extracellular traps differ from nuclear DNA‐based vital NETs in their composition and the signaling pathways involved. In this process, glutathionylation of cytoskeletal proteins, particularly actin and microtubules, regulates the structural rearrangements required for the catapult‐like expulsion of mtDNA [61]. Deficiency of glutaredoxin 1, the primary deglutathionylating enzyme, impairs cytoskeletal dynamics and prevents mtDNA release [62]. Mitochondria and glycolytic adenosine triphosphate (ATP) supply the energy for this process [63, 64].

In addition to ROS production, metabolic reprogramming is increasingly recognized as a prerequisite for NET formation. NET formation is strictly dependent on glucose through the glycolytic pathway. In vitro studies show that neutrophils stimulated in glucose‐free media undergo early chromatin decondensation but fail to complete NET release; restoration of glucose permits NET extrusion within minutes [65]. Treatment with the glycolysis inhibitor 2‐deoxyglucose completely abolishes NET release [66]. However, this dependence on glucose is not universal. Immature low‐density neutrophils can sustain NET formation under glucose‐deprived conditions by utilizing glutamate and proline catabolism for mitochondrial‐dependent ATP production [67]. Pentose phosphate pathway activity also increases during NET formation, supplying NADPH as a substrate for NADPH oxidase and thereby sustaining ROS generation [68, 69]. Recent work has further demonstrated that hypoxia‐inducible factor 1α (HIF‐1α)‐dependent glycolytic reprogramming enhances NET formation under hypoxic conditions, and that pharmacological inhibition of HIF‐1α or glucose uptake markedly reduces NET formation [70]. At the signaling level, the PI3K/Akt and ERK/JNK pathways serve as central switches. Akt promotes NET formation over apoptosis in a ROS‐dependent fashion [71], whereas JNK signaling contributes to the metabolic shift that licenses NET release [72]. Downstream of these metabolic and oxidative signals, neutrophil elastase (NE) and myeloperoxidase (MPO) function as key executors [73]. MPO facilitates NE release from azurophilic granules upon ROS exposure, and NE then translocates to the nucleus to partially degrade histones and drive chromatin decondensation [74, 75, 76]. These metabolic and signaling events are of particular relevance in AS, where hypoxic, glucose‐rich microenvironments within plaques may preferentially promote NET formation and sustain vascular inflammation [77].

### 3.2 PAD4 Activation, Chromatin Decondensation, and Nuclear Remodeling

PAD4 is the only nuclear‐localized member of the peptidylarginine deiminase family and is the principal enzyme responsible for histone citrullination during NET formation [78]. PAD4 converts arginine residues on histones H3 and H4 to citrulline, neutralizing their positive charge. This eliminates the electrostatic and hydrogen‐bond interactions between histones and DNA, leading to chromatin relaxation [79, 80]. PAD4 activity is strictly Ca^2^^+^ dependent. Elevated intracellular calcium induces conformational changes in the enzyme that expose its catalytic site, as demonstrated in vitro [81, 82, 83]. The downstream consequences of histone citrullination are not just simple charge neutralization. Citrullination of H3R8 generates a dual modification (H3Cit8K9me3) that displaces heterochromatin protein 1β (HP1β), promoting heterochromatin decondensation and enabling large‐scale chromatin unfolding [81]. High‐resolution time‐lapse microscopy of neutrophils undergoing NET formation has revealed a stereotyped sequence of nuclear events, including rapid actin cytoskeleton disassembly, microtubule and vimentin remodeling, nuclear lamin meshwork discontinuity, nuclear envelope rupture, DNA release into the cytoplasm, and finally plasma membrane rupture with extracellular chromatin discharge [83]. Importantly, both the citrullination activity and the nuclear localization signal of PAD4 are required for efficient NET formation, confirming that PAD4 must act within the nucleus to drive chromatin decondensation [83].

PAD4 does not act alone. Calpain, a Ca^2^^+^‐activated protease, cooperates with PAD4 by cleaving nuclear lamins and chromatin‐bound proteins [84]. Pharmacological or genetic inhibition of either PAD4 or calpain strongly blocks chromatin decondensation in both human and mouse neutrophils, indicating that citrullination licenses calpain‐mediated proteolysis at the nuclear envelope [85]. This cooperation ensures that chromatin decondensation is tightly coupled to nuclear lamina breakdown, allowing efficient NET extrusion. It should be noted that PAD4‐independent NET formation has been reported under specific stimuli, such as those engaging calcium ionophore pathways or certain NADPH oxidase‐dependent routes [86], suggesting that the requirement for PAD4 may be context dependent rather than absolute.

From a disease perspective, these chromatin remodeling events are particularly relevant in thrombo‐inflammatory settings. In atherosclerotic lesions, citrullinated histone H3‐positive NETs have been identified within both plaques and coronary thrombi, and PAD4 deficiency or pharmacological inhibition significantly delays arterial thrombosis in models of superficial erosion, although its effect on chronic plaque size appears limited [87]. These findings suggest that PAD4‐driven chromatin remodeling is not only a molecular hallmark of NET formation but also a potential therapeutic node in CVD.

### 3.3 Crosstalk Between NETosis, Apoptosis, and Pyroptosis

It is reported that lytic NET formation can be viewed as a distinct form of programmed necrotic cell death, which intersects with multiple regulated cell death pathways. Although traditionally regarded as a distinct death modality, NETosis shares key signaling nodes with apoptosis and pyroptosis, and the boundaries among these pathways are now recognized as context dependent. Rather than functioning as entirely separate programs, NETosis can be more accurately viewed as a distinct but partially overlapping cell death pathway that converges with apoptotic and pyroptotic signaling through shared effectors.

Apoptosis and NETosis are generally considered mutually exclusive outcomes of neutrophil activation [88]. ROS can initiate both processes, but the Akt and JNK pathways determine which pathway predominates [88]. **phorbol 12‐myristate 13‐acetate (PMA)**‐induced NADPH oxidase activation generates large amounts of cytosolic ROS, which activate Akt to promote NETosis while simultaneously suppressing apoptotic signaling [71, 89]. However, a study has challenged the traditional independence of these two pathways. Zhu et al. demonstrated that apoptotic signaling engages PAD4, establishing a cellular state primed for NETosis that occurs upon membrane disruption by gasdermin E (GSDME), suggesting that NETosis may represent a default secondary death program in apoptotic neutrophils that are not promptly cleared [78]. Autophagy represents another regulatory node. It can facilitate NETosis independently of ROS‐dependent mechanisms [90], and its interplay with lipid metabolism may further modulate the balance between NET formation and other death programs [91].

The relationship between gasdermin D (GSDMD) and NETosis has become a focus of active debate. Two landmark studies in 2018 reported that GSDMD is cleaved during NETosis and that its pore‐forming activity is required for NET release [92, 93]. In this model, Caspase‐11 activates GSDMD, which then permeabilizes both nuclear and plasma membranes, promotes NE release from azurophilic granules, and facilitates PAD4 activation by altering the cellular ion gradient [94]. However, this view has been challenged. Chauhan et al. demonstrated that PMA‐induced NETosis proceeds normally in GSDMD‐deficient neutrophils, although GSDMD remains important for canonical inflammasome‐driven pyroptosis [95]. More directly, Stojkov et al. showed that mouse neutrophils lacking GSDMD are fully competent in producing NETs in response to multiple physiological stimuli, including C5a, lipopolysaccharide (LPS), and canonical inflammasome activation, and that GSDMD is not cleaved during these responses [96]. These findings indicate that GSDMD is dispensable for NET formation under many physiological stimuli, though it may contribute in specific pathogen‐driven or noncanonical inflammasome contexts.

Furthermore, GSDME has recently been identified as a regulator of neutrophil pyroptosis [97]. Loss of GSDME specifically inhibits pyroptosis and redirects neutrophil death toward apoptosis [98]. Moreover, microvesicles from pyroptotic macrophages can transfer GSDMD‐N fragments and mitochondrial components to neutrophils, increasing intracellular ROS and accelerating NET formation, which in turn amplifies inflammation through a positive feedback loop [99]. A recent comprehensive review on regulated cell death in neutrophils has proposed that apoptosis, NETosis, and pyroptosis may converge on gasdermin activation as a common terminal effector, with the mode of death determined by the specific gasdermin member engaged and the threshold of pore formation achieved [88]. Together, these findings highlight a broader principle: NETosis, pyroptosis, and apoptosis are not isolated pathways but are connected through shared effectors including ROS, calcium flux, and gasdermin family members. The stimulus type, intracellular metabolic state, and tissue context collectively determine which pathway prevails in a given neutrophil. In the context of AS, where both sterile inflammation and metabolic stress coexist, this crosstalk may influence whether neutrophils undergo immunologically silent apoptosis or lytic NET release that amplifies plaque inflammation. Understanding these interconnections may inform the rational design of therapeutic strategies. For example, targeting PAD4 or gasdermin pores could selectively modulate NET formation, while preserving apoptotic clearance that promotes inflammation resolution. The relative contribution of each pathway in different stages of AS warrants further investigation.

## 4 Functional Architecture of NETs: Cooperative Actions of NET Components

The preceding sections established that NETs form through distinct molecular pathways. Once released, the biological impact of NETs depends not on any single component acting alone, but on the coordinated actions of their diverse constituents. This section adopts a mechanism‐oriented framework organized around four functional themes: (i) the DNA scaffold as a thrombo‐inflammatory platform, (ii) proteolytic and oxidative effectors, (iii) NET‐associated damage‐associated molecular patterns (DAMPs) and immune amplification, and (iv) the integrated, multitarget effects of these components on the atherosclerotic vessel wall. Throughout, we distinguish between evidence obtained directly from atherosclerotic tissues and supportive observations derived from thrombosis, stroke, or vascular injury models. A central concept throughout this discussion is that the NET scaffold concentrates and codelivers effector molecules at the endothelial surface, generating spatiotemporally organized, high‐density microenvironments that are unlikely to be achieved by soluble mediators diffusing freely in the circulation.

### 4.1 Structural Scaffolds and Thrombo‐Inflammatory Platforms

**FIGURE 3:**
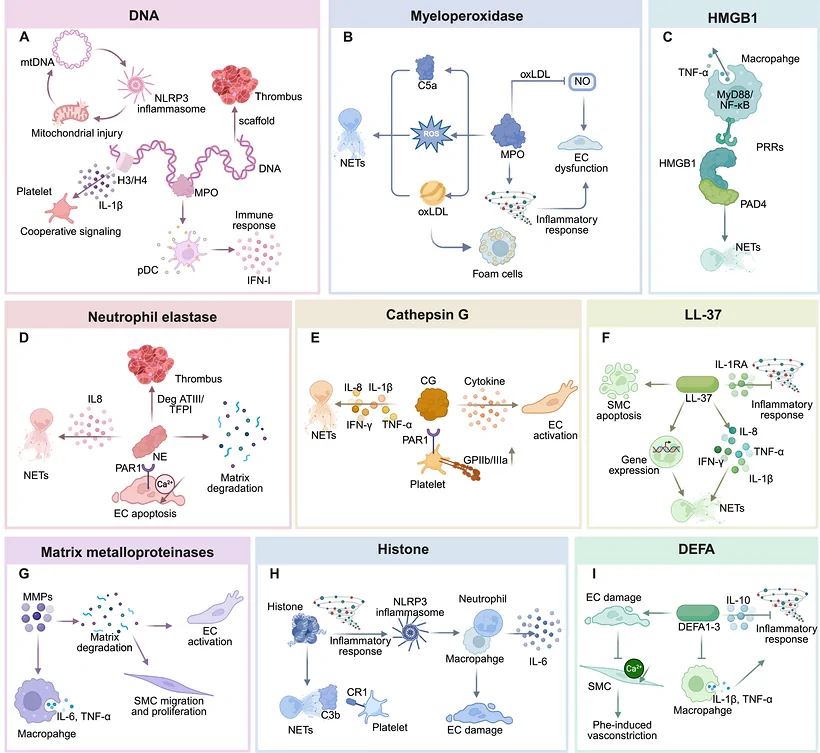
Contributions of NET‐derived components to atherosclerosis. NETs promote vascular inflammation, thrombosis, and matrix remodeling through distinct effectors. (A) DNA activates cGAS–STING pathway and AIM2 inflammasomes, triggering Type I interferon responses. (B) MPO oxidizes LDL, generates ROS, and reduces endothelial NO, driving foam cell formation. (C) HMGB1 stimulates macrophage NF‐κB signaling and sustains NET‐driven inflammation. (D) NE degrades anticoagulants, induces endothelial apoptosis, and remodels the extracellular matrix. (E) Cathepsin G activates platelets and processes cytokines, enhancing endothelial activation. (F) LL‐37 induces smooth muscle cell apoptosis and inflammatory signaling, and stabilizes NETs. (G) MMPs degrade matrix, activate endothelium, and promote smooth muscle migration. (H) Histones trigger endothelial damage, NLRP3 inflammasome activation, and IL‐6 release. (I) DEFA1‐3 damage endothelium, modulate macrophages, and affect vascular tone. *Created with BioRender.com. CG, cathepsin G; cGAS, cyclic GMP–AMP synthase; DEFA, defensins; EC, endothelial cell; HMGB1, high‐mobility group box 1; IL, interleukin; LDL, low‐density lipoprotein; MMP, matrix metalloproteinase; MPO, myeloperoxidase; NE, neutrophil elastase; NET, neutrophil extracellular trap; NF‐κB, nuclear factor kappa‐light‐chain‐enhancer of activated B cells; PAR, protease‐activated receptor; pDC, plasmacytoid dendritic cell; ROS, reactive oxygen species; SMC, smooth muscle cell; TFPI, tissue factor pathway inhibitor.

The DNA backbone of NETs is not a passive structural element. It provides a spatially organized surface on which prothrombotic and proinflammatory molecules concentrate, and this colocalization is central to the pathogenic potency of NETs (Figure 3A). DNA itself has limited direct cytotoxicity toward endothelial cells (ECs). Its primary contribution lies in binding and concentrating procoagulant molecules, including von Willebrand factor (VWF), fibrinogen, fibronectin, factor XII, and tissue factor, at a single interface [100]. By anchoring these factors in close proximity, the NET scaffold physically entraps platelets, red blood cells, and other circulating cells within the developing thrombus. Much of this evidence comes from in vitro reconstitution experiments and venous thrombosis models [101]. However, the detection of NET–fibrin composites in coronary thrombi from patients with acute myocardial infarction(AMI) supports the relevance of this scaffold function in arterial settings [102]. The scaffold also enables cooperative signaling between its constituents. Histones H3 and H4 decorate the DNA mesh and promote platelet activation through complement C3b deposited on NETs, which engages complement receptor 1 receptors on platelets [103]. Histones and DNA together activate Toll‐like receptor 4 (TLR4) more efficiently than either component alone, likely because their copresentation on the scaffold facilitates receptor engagement [104]. This cooperativity illustrates a general principle. The NET scaffold codelivers ligands in spatial configurations that amplify downstream signaling beyond what individual soluble components typically achieve. Moreover, the resistance of intact NETs to rapid enzymatic degradation confers temporal persistence, allowing these spatially organized signals to act over hours rather than the minutes characteristic of soluble mediator bursts.

Unlike nuclear DNA, mtDNA carries a bacteria‐like hypomethylation pattern recognized by the innate immune system as a DAMP [105]. Both native and oxidized mtDNA induce NET formation, and the resulting NLRP3 inflammasome activation causes further mitochondrial injury, releasing additional damaged mtDNA in a positive feedback loop [106]. In atherosclerotic tissues specifically, mtDNA damage correlates with lesion burden and, in human cohort studies, precedes the clinical onset of disease [105, 107, 108, 109, 110], raising the possibility that mtDNA‐driven inflammation is involved in early atherogenesis. The proposed downstream mechanisms include the accumulation of monocytes laden with damaged mtDNA within the plaque microenvironment [111, 112, 113].

Extracellular DNA also forms immune complexes with MPO and Proteinase 3 [114]. These complexes bind to Fcγ receptors on plasmacytoid dendritic cells (pDCs), leading to TLR9‐dependent pDC activation and a Type I interferon (IFN‐I) response [99, 115, 116]. This pathway has been demonstrated in murine AS models, where antibody‐mediated depletion of pDCs reduced plaque burden and IFN‐I activity [49, 117], providing evidence for its atherogenic relevance. NETs also exert prothrombotic effects through the coreleased enzyme PAD4. Once in the circulation, PAD4 citrullinates ADAMTS13, the metalloprotease that cleaves ultra‐large VWF multimers. Citrullination markedly inhibits ADAMTS13 enzymatic activity, causing VWF–platelet strings to persist on the endothelial surface and accelerating thrombus formation after vessel injury in mice [118, 119]. Although this mechanism was established in mesenteric venule injury models and in plasma from septic patients, it may have relevance to atherothrombotic settings, where both elevated PAD4 activity and reduced ADAMTS13 function have been observed. In patients with ST‐elevation myocardial infarction (STEMI), elevated double‐stranded DNA (dsDNA) levels correlate with platelet aggregation, and mechanistic studies have implicated the cGAS–STING/NLRP3/Caspase‐1/IL‐1β axis in platelets [120]. In the cerebrovascular system, NET‐derived DNA triggers the AIM2 inflammasome in vulnerable atherosclerotic plaques, promoting destabilization and atherothrombosis [121]. These clinical observations, while correlative, are consistent with the scaffold‐mediated prothrombotic mechanisms characterized in experimental systems.

### 4.2 Proteolytic and Oxidative Effectors

NETs carry a concentrated payload of proteases and oxidative enzymes that degrade the extracellular matrix (ECM), impair anticoagulant defenses, and amplify coagulation. This spatial concentration is especially relevant in AS, where NETs deposit directly onto the plaque endothelium.

NE and cathepsin G (CG) coordinate serine protease activity (Figure 3D, E). NE consists of 218 amino acid residues arranged in two β‐barrel domains [122]. It degrades key anticoagulant factors such as antithrombin III and tissue factor pathway inhibitor (TFPI), shifting hemostatic balance toward coagulation [123, 124]. Simultaneously, NE activates MMP‐2 and MMP‐9, while inactivating TIMP‐1 and accelerating ECM degradation [125]. CG complements these actions by independently degrading TFPI and promoting fibrin formation [126], and by processing processing protease‐activated receptor 4 to drive platelet shape change and Glycoprotein IIb–IIIa expression [73, 127]. In atherosclerotic lesions specifically, CG embedded in NETs cleaves IL‐1α precursors on the intimal surface, releasing the mature cytokine and amplifying EC activation [128]. This activity parallels the function of Caspase‐1 in inflammasome‐dependent IL‐1β maturation and may represent a mechanism through which NET‐bound CG preferentially activates ECs, given that the scaffold concentrates the protease in proximity to its endothelial substrates. Within NETs, NE also promotes the endothelial‐to‐mesenchymal transition through the β‐catenin signaling pathway and cleaves PAR‐1 to induce EC apoptosis. Evidence that NE disrupts EC barrier integrity by degrading VE‐cadherin and the actin cytoskeleton has been obtained in pulmonary vascular injury models [129, 130]. While these observations were not made in atherosclerotic arteries, they point to conserved mechanisms of NE‐mediated endothelial damage whose relevance to plaque erosion merits further study.

MPO drives oxidative modification at the scaffold surface (Figure 3B). MPO, a heme‐containing glycosylated tetramer closely associated with NET release [131], generates hypochlorous acid that oxidizes LDL to oxLDL, thereby promoting foam cell formation [132, 133]. In human atherosclerotic plaques, MPO‐mediated tyrosine chlorination impairs cholesterol efflux from lesion macrophages [134]. MPO also oxidizes endothelial nitric oxide synthase, reducing nitric oxide bioavailability and compounding endothelial dysfunction through accumulation of asymmetric dimethylarginine [135]. In addition to its direct enzymatic effects, MPO participates in an amplification circuit linked to NET persistence. Complement activation within atherosclerotic plaques generates C5a, which binds to C5aR1 on neutrophils and potently induces further NET formation and MPO release [136]. This creates a positive feedback loop in which NET‐driven inflammation sustains complement activation, perpetuating local inflammation. C5a has also been causally linked to plaque disruption and intraplaque hemorrhage in murine models of advanced AS [137].

Matrix metalloproteinases (MMPs) are externalized via a scaffold‐mediated mechanism (Figure 3G). MMP‐2, MMP‐9, and MMP‐8 degrade vascular matrix collagen and contribute to fibrous cap thinning [138]. MMP‐9 content and activity are elevated in human atherosclerotic plaques [138, 139], and during NET formation, intracellular MMP‐9 is externalized by neutrophils and deposited directly onto the surrounding tissue [140]. This externalization mechanism is distinct from macrophage‐derived MMP secretion, which occurs via classical exocytosis into the interstitial space. The scaffold‐directed delivery of MMP‐9 tends to concentrate proteolytic activity at the NET–endothelium interface, potentially enhancing local matrix degradation relative to diffuse secretion [141]. ECs, smooth muscle cells (SMCs), and macrophages within human plaques can also express MMP‐8, suggesting that NET‐derived MMPs may act in concert with cell‐autonomous MMP production to weaken the fibrous cap [142].

The functional synergy among these effectors is a notable feature of NET‐mediated vascular injury. NE activates latent MMPs; MPO‐generated oxidants modify protease substrates, rendering them more susceptible to cleavage; and the scaffold colocalizes these enzymes at the endothelial surface. This layered proteolytic–oxidative program is expected to produce greater local tissue damage than any single enzyme acting in isolation and may help account for the distinct pathological contribution of NETs compared with the more diffuse proteolysis associated with macrophage degranulation.

### 4.3 NET‐Associated DAMPs and Immune Amplification

NETs serve as potent activators of innate and adaptive immunity through the release of DAMPs. Three NET‐associated danger signals are particularly relevant to AS: histones, high‐mobility group box 1 (HMGB1), and LL‐37. Each engages pattern recognition receptors and sustains inflammatory cascades, but their pathogenic significance in AS varies with the strength of available evidence.

Histones are the most abundant protein component of NETs, forming an octamer of H2A, H2B, H3, and H4 [143] (Figure 3H). In atherosclerotic lesions, extracellular histones function as DAMPs that trigger oxidative stress and NLRP3 inflammasome activation, recruiting neutrophils and macrophages and driving IL‐6 and other proinflammatory cytokine secretion [144]. Histone citrullination by PAD4 enhances nucleosome binding to TLR4, amplifying proinflammatory signaling [104]. A feature that may distinguish histone‐mediated inflammation in the context of NETs from the generic consequences of cell death is the manner of delivery. The NET scaffold presents histones in a stable, locally concentrated array that may prolong receptor engagement at the vessel wall, in contrast to the more transient release of histones during apoptosis or necrosis.

HMGB1, a DNA‐binding protein contained in NETs, signals through Toll‐like receptor 2 (TLR2), TLR4, and the receptor for advanced glycation end products on macrophages, activating the myeloid differentiation primary response 88/nuclear factor κB pathway and inducing tumor necrosis factor α secretion [145, 146] (Figure 3C). HMGB1 also interacts with PAD4, promoting additional NET formation and thrombosis [147]. Current evidence linking HMGB1 to NET‐mediated cardiovascular pathology comes primarily from ischemia–reperfusion models, including a rat stroke model in which HMGB1 mediated NET‐driven neuronal death [148, 149] and a murine heart failure model in which it promoted neutrophil recruitment [150], rather than from chronic atherosclerotic lesion studies. These observations support a role for HMGB1 in sustaining NET‐mediated inflammatory cycles in cardiovascular tissues, although its specific contribution to plaque biology warrants further investigation.

LL‐37, a human cathelicidin, has a distinctive dual role in NET biology (Figure 3F). It stimulates neutrophils to form additional NETs in a dose‐dependent manner and binds to existing NETs to protect them from DNase degradation [151, 152]. This combination of NET induction and NET stabilization prolongs the persistence of NETs at inflammatory sites. LL‐37 also recruits neutrophils by upregulating IL‐8 and MCP‐1 [153]. In atherosclerotic plaques specifically, LL‐37 mediates SMC apoptosis, which slows fibrous cap formation and accelerates plaque vulnerability [49, 154]. LL‐37 has also been shown to potentially enhance thrombin activity [155].

In addition to the major NET‐associated DAMPs discussed above, neutrophil α‐defensins may exert context‐dependent effects in AS (Figure 3I). Studies have linked α‐defensins to endothelial dysfunction [156], vascular retention of atherogenic lipoproteins [157], and acute coronary syndromes [158]. However, most of this evidence comes from earlier vascular studies or clinical association analyses rather than from plaque‐focused investigations that directly define the contribution of NET‐associated α‐defensins within atherosclerotic lesions. Some findings also suggest that α‐defensins may exert protective effects under specific conditions, indicating that their role is not uniformly pathogenic [159, 160]. Taken together, the current evidence supports a bidirectional and context‐dependent role for α‐defensins in AS.

The common feature linking these DAMPs is their capacity to establish positive feedback loops that are sustained over time. NET‐derived DAMPs activate innate immune cells, which release cytokines and chemokines that recruit additional neutrophils, promote further NETosis, and generate more DAMPs. This prolonged, localized immune activation is unlikely to resolve without disruption of the NET structure by endogenous DNases or therapeutic intervention.

### 4.4 Multicomponent, Multitarget Coupling: NETs as Integrated Effectors of Plaque Destabilization

The mechanisms described in Sections 4.1–4.3 converge on the atherosclerotic vessel wall. Rather than recapitulating individual pathways, this section highlights the emergent properties that arise when multiple NET components act simultaneously on multiple cell types. These properties may position NETs as integrated effectors of plaque destabilization and atherothrombosis. The first property is simultaneous multitarget engagement. As an activated or eroding plaque, a single NET contacts ECs from the luminal side, entraps platelets on VWF strings, and exposes its DAMP content to subendothelial immune cells. The physical dimensions of the NET scaffold, spanning tens of micrometers, bridge the endothelial surface and plaque stroma, creating a continuous zone of high effector density where the proteolytic, oxidative, and immunostimulatory activities operate in parallel rather than in sequence. The second emergent property is bidirectional amplification with macrophages. Within the plaque microenvironment, NETs and macrophages amplify each other through several converging pathways, with cholesterol crystals and oxLDL driving reciprocal NET–macrophage activation loops [161]. This reciprocal amplification establishes a self‐sustaining inflammatory circuit within the plaque that is qualitatively different from unidirectional cytokine signaling. The third emergent property is direct SMC cytotoxicity and cap weakening. Unlike macrophage‐derived mediators mainly modulate SMC phenotype switching, whereas NET components kill SMCs directly [162]. Histone H4 induces rapid, receptor‐independent SMC death through membrane pore formation, and neutralization of H4 significantly improves SMC viability [163]. LL‐37 contributes to SMC loss through a distinct apoptotic mechanism [154]. Simultaneously, NET‐associated NE and MMPs degrade the collagen matrix that SMCs produce. This combined assault, killing the cells that maintain the fibrous cap while degrading the matrix they have already deposited, making it a particularly effective mode of plaque destabilization. Evidence for this mechanism comes from studies of human carotid plaques, in which NETs colocalized with regions of fibrous cap thinning and SMC loss [87]. When the EC barrier fails, the NET scaffold becomes the immediate interface between blood and the exposed plaque. The prothrombotic activities described above then converge on this single scaffold. Histones activate platelets, serine proteases degrade anticoagulant factors, and NET DNA recruits coagulation factors, making NETs a particularly efficient platform for initiating and propagating thrombus formation on disrupted plaques. PAD4 deficiency selectively reduces acute thrombotic complications without altering chronic plaque size, providing experimental evidence for a stage‐specific, NET‐dependent contribution to atherothrombosis [87]. Taken together, the functional architecture generates an integrated effector system whose combined vascular impact exceeds what any individual component or pathway would produce in isolation. Future studies are needed to determine whether this integrated mode of action can be exploited therapeutically and to identify which cooperative interactions offer the most tractable targets for intervention in human atherosclerotic disease.

## 5 NETs in the Pathological Continuum of AS and CVD

**FIGURE 4:**
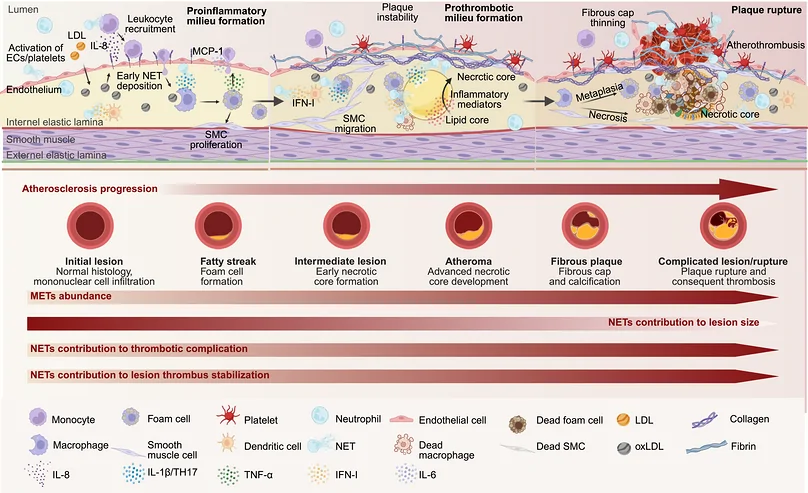
Role of neutrophil extracellular traps (NETs) in the pathogenesis and progression of atherosclerosis. This figure illustrates the major processes driven by NETs, although in reality these mechanisms form a continuous, overlapping sequence. NETs act in a stage‐specific manner during atherosclerotic cardiovascular disease. In early atherogenesis, DNA–protein complexes released from NETs activate plasmacytoid dendritic cells via TLR9, inducing Type I interferon responses and promoting monocyte recruitment, which amplifies local vascular inflammation. As plaques progress, NETs and macrophages engage in a reciprocal loop mediated by IL‐1β and miR‐146a, sustaining inflammation and driving plaque growth. In advanced lesions, NETs provide immunothrombotic scaffolds that concentrate tissue factor and von Willebrand factor, while NET‐associated proteases including NE, CG, and MMPs degrade the fibrous cap, facilitating plaque rupture or erosion and subsequent thrombus formation. Through these integrated and overlapping actions, NETs serve as central effectors linking inflammation, vascular injury, and thrombosis. *Created with BioRender.com. CG, cathepsin G; EC, endothelial cell; IFN‐I, Type I interferon; IL, interleukin; LDL, low‐density lipoprotein; METs, macrophage extracellular trap; MMP, matrix metalloproteinase; NE, neutrophil elastase; NET, neutrophil extracellular trap; pDC, plasmacytoid dendritic cell; SMC, smooth muscle cell.

**FIGURE 5:**
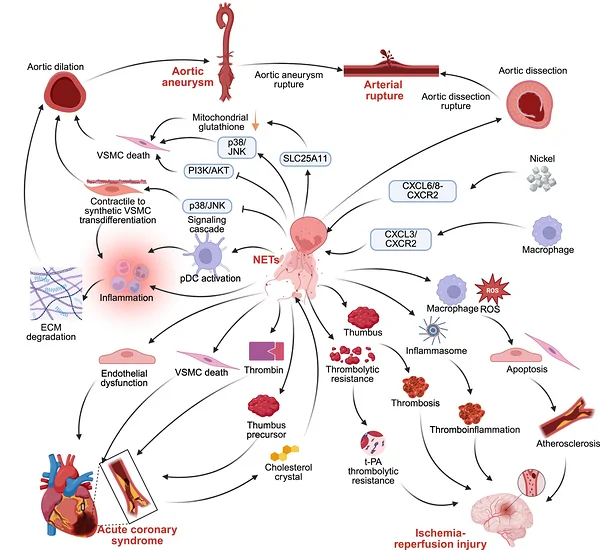
Neutrophil extracellular traps (NETs) in various cardiovascular diseases. NETs are key drivers of aortic wall structural degradation. By inducing vascular smooth muscle cell (VSMC) death, promoting their phenotypic transdifferentiation from a contractile to a synthetic state, exacerbating local inflammation, and directly degrading the extracellular matrix (ECM), they collectively compromise the integrity of the aortic wall. This cascade of pathological changes is the core driver of aortic dilation, aneurysm formation, aortic dissection, and ultimately, arterial rupture. NETs play a multifaceted role in the pathogenesis of acute coronary syndrome. They drive the progression and instability of atherosclerotic plaques by serving as the core scaffold for intracoronary thrombus formation. This, coupled with NET‐induced endothelial dysfunction and VSMC death, forms the pathological basis of acute coronary events. Following the restoration of blood flow to ischemic organs such as the heart or brain, NETs act as key inflammatory mediators that exacerbate tissue damage. During the reperfusion phase, abundantly formed NETs, which are resistant to tissue‐type plasminogen activator (t‐PA)‐mediated thrombolysis, can occlude the microvasculature and dramatically amplify the local inflammatory storm and oxidative stress, leading to apoptosis and necrosis, thereby significantly increasing the final infarct size. *Created with BioRender.com. CXCL, C–X–C motif chemokine ligand; CXCR, C–X–C motif chemokine receptor; ECM, extracellular matrix; NET, neutrophil extracellular trap; pDC, plasmacytoid dendritic cell; ROS, reactive oxygen species; SLC25A11, solute carrier family 25 member 11; t‐PA, tissue‐type plasminogen activator; VSMC, vascular smooth muscle cell.

AS and its cardiovascular complications form a pathological continuum rather than a collection of separate diseases (Figures 4 and 5). Early lipid‐driven inflammation, plaque destabilization, acute thrombotic occlusion, and ischemia–reperfusion injury represent successive stages in which vascular damage accumulates progressively. NETs participate throughout this continuum, but their dominant function shifts with disease stage. In early atherogenesis, they mainly amplify local inflammation and sustain endothelial dysfunction. As plaques become vulnerable, they serve as structural scaffolds for immunothrombosis. After reperfusion, they obstruct the microvasculature and drive adverse cardiac remodeling. Whether NETs exert a net pathological or protective effect at any given stage depends on several factors. These include release intensity, aggregation state, clearance efficiency, the local microenvironment, and the temporal phase of disease. This section follows the natural history of atherosclerotic CVD to examine the stage‐specific roles of NETs.

### 5.1 NETs in Atherogenesis and Plaque Progression

Histological studies have confirmed the presence of NETs within atherosclerotic lesions in both humans and mice [164]. Neutrophils and NETs have also been identified in thrombi aspirated from patients with AMI [111]. Together, these findings position NETs as active contributors to atherosclerotic disease rather than incidental byproducts of neutrophil activation. Within the atherosclerotic microenvironment, several feed‐forward loops sustain NET formation. Cholesterol crystals stimulate vascular smooth muscle cells to release IL‐33, which enhances neutrophil adhesion, ROS production, and NET generation [165]. The resulting NETs engage the reciprocal NET–macrophage amplification loop described in Section 4.4 [28, 166]. Genetic factors modulate this interplay as well. Low‐frequency *Jak2V617F* mutations accelerate AS by enhancing IL‐1β signaling, promoting cleavage of macrophage phagocytic receptors, and fostering plaque instability [167]. Recent studies have expanded the mechanistic landscape beyond soluble mediators. Combining bioinformatics with tissue‐level validation, Cao et al. identified MD‐1/LY86 as an upstream inducer of NETs in unstable carotid plaques [168]. This finding reveals a new node connecting the plaque microenvironment to NET generation. Biomechanical cues contribute as well. Low shear stress, a hallmark of AS‐prone vascular regions, exacerbates NET formation through Piezo1‐mediated mechanosensation [169]. These findings indicate that physical forces, not only biochemical signals, regulate NET activity during atherogenesis. Many of the individual effects attributed to NETs, such as endothelial injury, cytokine amplification, and lipid oxidation, are also shared by macrophages, platelets, and adaptive immune cells. Compared with these individual mediators, however, NETs are structurally distinct in that they simultaneously deliver proteolytic enzymes, cytotoxic histones, and immunostimulatory DNA within a single extracellular scaffold [52]. This structural integration enables NETs to coordinate multiple pathogenic processes at the same site. Whether this coordination makes NETs quantitatively dominant over other inflammatory mediators in atherogenesis remains an open question. Nevertheless, interventional studies demonstrate that NETs are nonredundant contributors to atherogenesis. DNase I treatment and pharmacological PAD4 inhibition with Cl‐amidine attenuate plaque burden and inflammation in murine models [76, 143, 170, 171]. Hematopoietic PAD4 deficiency alone, however, did not significantly alter chronic plaque size in hypercholesterolemic mice, even though it reduced arterial thrombosis [87]. These findings suggest that NET‐mediated effects on atherogenesis may involve both PAD4‐dependent and PAD4‐independent mechanisms.

Interestingly, NETs are not uniformly harmful in the context of AS. When NETs aggregate at high local density, the resulting aggregated NETs (aggNETs) can degrade proinflammatory cytokines and chemokines through the proteolytic activity of NET‐bound serine proteases, thereby dampening rather than amplifying inflammation [7]. Whether this regulatory mechanism operates in atherosclerotic plaques in vivo has not been confirmed, but it illustrates a general principle. The biological outcome of NET formation depends on their release intensity, aggregation state, local clearance efficiency, and the specific microenvironmental context. This complexity cautions against treating NET formation in AS as a uniformly deleterious process.

### 5.2 NETs in Plaque Destabilization and Acute Thrombotic Events

The progression from stable AS to acute cardiovascular events marks as a critical shift, in which the role of NETs moves from inflammatory amplification to immunothrombotic scaffold assembly. When coronary AS advances sufficiently, rupture or erosion of vulnerable plaques triggers secondary thrombosis, the principal pathological basis of acute coronary syndrome (ACS) [172] (Figure 4). Three mechanisms commonly lead to coronary thrombosis: plaque rupture, plaque erosion, and nodule calcification [173]. Patients with ACS typically show increased neutrophil counts and dysregulated immune activation, providing a permissive environment for NET generation. Clinical evidence consistently associates NETs with the severity of acute coronary events. Thrombi extracted from the coronary vasculature of ACS patients contain significantly higher levels of NET biomarkers than those of healthy controls [102]. In STEMI patients, greater NET burden and higher DNase I activity within thrombi correlate with larger infarct size and delayed ST‐segment resolution [174]. Rising serum dsDNA concentrations correlate with microvascular obstruction in STEMI patients. NETs have also been identified in 25% of in‐stent thrombosis samples after percutaneous coronary intervention (PCI), where they serve as a marker of stent thrombosis [175, 176]. Mechanistically, NETs promote thrombosis through the converging scaffold‐mediated, proteolytic, and coagulation‐activating mechanisms [101, 102]. Conventional thrombosis is driven primarily by vascular injury, whereas immunothrombosis incorporates innate immune cells and their products as integral thrombus components. Within this process, NETs activate both the intrinsic and extrinsic coagulation pathways simultaneously [52]. The broader relevance of this paradigm became apparent during the COVID‐19 pandemic, in which excessive NET formation drove widespread microvascular thrombosis and correlated with disease severity [177]. The colocalization of citrullinated histone H3 with platelets within pulmonary microthrombi underscores the pathogenic interplay between NETs and platelets during viral infection [178]. Together, these observations generalize the concept that dysregulated NET formation drives thrombotic complications across diverse clinical settings, from atherosclerotic plaque disruption to infection‐associated coagulopathy.

A key emerging insight is that NETs contribute to acute thrombosis through different mechanisms in plaque rupture versus plaque erosion, and this distinction has direct therapeutic implications. In plaque rupture, the breach of the fibrous cap exposes the lipid‐rich necrotic core to the bloodstream. The released cholesterol crystals upregulate proinflammatory cytokines, recruit polymorphonuclear neutrophils (PMNs), and induce NET formation. Active tissue factor on NETs promotes thrombus formation at the ruptured plaques and activates platelets through the PAR signaling, driving thrombus enlargement [28]. Here, NETs function mainly as amplifiers of a coagulation cascade already initiated by the exposed necrotic core. Plaque erosion differs fundamentally because the fibrous cap remains intact. Erosion is characterized by EC stripping from the plaque surface with mild luminal stenosis and accounts for approximately 31% of ACS cases [179]. In eroded plaques, NETs appear to act as primary initiators rather than secondary amplifiers. They induce oxidative stress and EC denudation through TLR2 signaling and secrete IL‐8 to recruit additional PMNs. This establishes a positive feedback loop that amplifies endothelial loss and promotes thrombosis on an otherwise structurally intact plaque [129, 180]. PMNs and NETs are abundant in all culprit plaques of patients with AMI, regardless of whether the mechanism is rupture, erosion, or intraplaque hemorrhage [180]. The proportional contribution of NETs to the thrombotic trigger is nonetheless likely greater in erosion, where conventional rupture‐based mechanisms are absent. This rupture–erosion distinction carries practical therapeutic weight. The 2018 EROSION study demonstrated that in ACS caused by plaque erosion with nonflow‐limiting stenosis, intensive dual antiplatelet therapy is a viable alternative to routine stenting [181]. Targeting NETs and their components may therefore offer a particularly rational approach for erosion‐mediated ACS, where the NET–EC–PMN axis is the primary driver of thrombosis. New antithrombotic approaches targeting the NET scaffold may complement existing therapies. DNase I can accelerate thrombus dissolution by degrading the DNA backbone of NETs without increasing bleeding risk, offering a potential strategy for secondary prevention of coronary heart disease and postinfarction management.

### 5.3 NETs in Ischemia–Reperfusion Injury and Postinfarction Remodeling

The restoration of blood flow following myocardial ischemia is essential for tissue salvage, but reperfusion itself paradoxically exacerbates injury. At this stage, the dominant function of NETs shifts again, from scaffolding thrombosis to obstructing the microvasculature and intensifying the local inflammatory response. In a rat model of acute myocardial infarction, Ge et al. [182] demonstrated that combined treatment with DNase I and r‐tPA reduced NET density, decreased the no‐reflow area, and limited infarct size relative to untreated controls. Long‐term follow‐up confirmed that this regimen also improved postinfarction left ventricular remodeling. Savchenko et al. [183] replicated these findings in mice, demonstrating significant cardioprotection with DNase I. Notably, PAD4‐null mice, which cannot produce NETs, were substantially protected from myocardial ischemia–reperfusion injury, and DNase I conferred no additional benefit in these animals. This genetic evidence supports a causal role for NETs in reperfusion injury rather than a mere correlative association.

In the anoxic microcirculation, NETs provide a scaffold for microthrombosis and aggravate endothelial injury, two key features of the coronary no‐reflow phenomenon [182]. Neutrophils recruited to the ischemic myocardium release large amounts of ROS through NADPH oxidase‐mediated respiratory bursts, directly damaging host tissues by modifying amino acids, proteins, and lipids [184]. Moderate ROS production supports cardiac repair, but excessive generation is harmful and promotes further tissue destruction. After the acute phase, NETs contribute to adverse remodeling. In STEMI patients, fibroblasts accumulate at infarcted sites and within coronary thrombi, and the local NET burden correlates with fibroblast activation and left ventricular dysfunction [185]. NETs have been shown to promote monocyte differentiation and induce fibroblast activation [186]. These data suggest that NETs mediate fibrotic remodeling after STEMI by stimulating fibroblasts. Their pathological impact thus extends well beyond the acute thrombotic event, into the chronic structural changes that underlie heart failure.

NETs may also contribute to repair in the postinfarction setting. In the infarcted myocardium, the neutrophil secretome, including NET‐derived components such as S100A8/A9, has been reported to promote macrophage polarization toward the M2 reparative phenotype [187]. N2‐phenotype neutrophils, which predominate in the later repair phase (approximately Days 4–7 post‐MI), release factors such as TGF‐β, and IL‐10 that support anti‐inflammatory macrophage responses [187]. Several caveats apply, however. Most evidence for NET‐mediated reparative effects comes from in vitro or early‐stage in vivo studies, and the extent to which this occurs in human postinfarction hearts remains unclear. The reparative function appears to be strictly time dependent [188]. The same NET components that support repair during the resolution phase may worsen inflammation if released during the early acute phase [186, 189]. The balance between profibrotic and proreparative effects on fibroblasts probably varies with infarct size, patient comorbidities, and concurrent therapies [190, 191]. These observations support a phase‐dependent dual role for NETs in postinfarction biology, but the evidence for their reparative function is considerably thinner than that for their pathological effects.

In summary, the evidence reviewed in this section demonstrates that NETs function as a common mechanistic thread linking the progressive stages of atherosclerotic CVD, with their dominant pathological contribution shifting as lesions evolve. A consistent theme across all stages is that the net biological effect depends on the interplay among release intensity, clearance efficiency, and the local microenvironment. These considerations argue for stage‐specific rather than blanket approaches to NET‐targeted cardiovascular therapy.

## 6 Clinical and Translational Evidence Linking NETs to Cardiovascular Outcomes

Human evidence increasingly supports NETs as both markers and mediators of coronary thrombo‐inflammation. Yet the clinical data remain largely observational, and no NET‐associated marker has been validated as a standalone biomarker for routine cardiovascular risk assessment. Interpreting this evidence requires attention to the biological specificity of each marker, the anatomical sampling site, the timing of measurement, and the disease context. At present, the strongest data link NET‐associated markers to the local thrombo‐inflammatory burden in acute and chronic coronary syndromes. Whether this association can be translated into clinically actionable information is an unresolved question.

### 6.1 Circulating NET Biomarkers and Clinical Correlations

The clinical value of any circulating NET marker is inseparable from its biological specificity, and the published literature has not always drawn this distinction with sufficient care. Cell‐free DNA (cfDNA) and dsDNA are released during apoptosis, necrosis, and NETosis alike, so they reflect general tissue injury rather than NET formation per se. MPO and NE reside in neutrophil granules and are discharged during degranulation independently of chromatin externalization; they are neutrophil‐associated but not NET‐specific. MPO–DNA complexes and citrullinated histone H3 more closely mirror the chromatin decondensation that defines NETs, and they are currently regarded as the most NET‐enriched circulating markers available. An elevated dsDNA level in a patient with myocardial infarction may reflect myocardial necrosis, neutrophil activation, NETosis, or any combination of these processes.

Several cohort studies link NET‐associated markers to the burden of coronary AS. In a cross‐sectional cohort of 282 patients with suspected coronary artery disease (CAD), Borissoff et al. found that plasma dsDNA, nucleosomes, citrullinated histone H4, and MPO–DNA complexes were each associated with coronary stenosis severity and elevated thrombin–antithrombin levels [51]. Nucleosome concentrations were an independent risk factor for severe stenosis, and MPO–DNA complexes correlated with multivessel disease. The cross‐sectional design, however, could not resolve whether elevated markers drive plaque progression or simply accompany advanced disease. Where blood is sampled substantially affects what the markers reveal. In a prospective STEMI trial (NCT01777750), Mangold et al. showed that dsDNA, CitH3, nucleosomes, MPO, and NE were all markedly elevated at the coronary culprit site relative to peripheral blood [192]. High culprit site dsDNA and neutrophil counts correlated with greater microvascular obstruction and lower ejection fraction, while higher local DNase activity tracked with less microvascular obstruction and better systolic function. These gradients indicate that the local equilibrium between NET accumulation and enzymatic clearance may influence tissue perfusion after reperfusion, although observational sampling alone cannot establish causality. Moreover, a recurring finding in this literature is that less specific general‐injury markers tend to show stronger and more reproducible clinical associations than more NET‐enriched markers. In a large cohort of 956 STEMI patients followed for a median of 4.6 years, Langseth et al. found that elevated serum dsDNA was independently associated with mortality after adjustment for conventional risk factors [193]. In the same cohort, however, the more NET‐specific markers CitH3 and MPO–DNA showed no significant association with the primary composite endpoint. This dissociation illustrates a core tension in NET biomarker research. The broad sensitivity of dsDNA, which captures myocardial necrosis, neutrophil death, and NETosis simultaneously, is exactly what prevents attribution of the prognostic signal to NETs alone. The failure of CitH3 and MPO–DNA to predict outcomes peripherally, in turn, may reflect their rapid proteolytic clearance in plasma, suboptimal assay sensitivity at the time of sampling, or a genuine disconnect between peripheral NET‐specific markers and the local NET burden within coronary thrombi. Measurement timing compounds these difficulties. MPO has been linked to 30‐day ischemic risk in some studies, but this association attenuates by 6 months, indicating that marker kinetics and the measurement window interact to shape prognostic performance. A recent study by Blasco et al. in STEMI patients reinforced this paradox [194]. Circulating levels of the NET‐specific marker nucleosome CitH3‐DNA at the time of primary PCI were not associated with 30‐day major cardiovascular events. By contrast, classical inflammatory markers such as C‐reactive protein (CRP) and IL‐6 added significant discriminative value to clinical prediction models. This finding does not negate the pathophysiological relevance of NETosis in coronary thrombosis, but it does caution against equating biological involvement with biomarker utility. Local or pathway‐specific markers may prove more informative than generic peripheral measures. Zhang et al. reported that plasma S100A12, an upstream trigger of NET formation, correlated with dsDNA concentrations in STEMI patients, and that higher levels of both were associated with increased 1‐year all‐cause mortality [140]. In a separate AMI cohort, NE levels in the infarct‐related artery independently predicted cardiovascular events [195]. Together, these data suggest that local or pathway‐specific markers may outperform generic peripheral measures in the acute coronary thrombosis setting, although external validation is needed before clinical application can be considered.

Elevated NET‐associated markers have also been reported in disease contexts where their biological meaning differs from that in acute coronary thrombosis. In acute aortic dissection, circulating CitH3, cfDNA, and nucleosomes likely reflect vascular wall injury and systemic inflammatory activation [196]. In atrial fibrillation, NET markers have been associated with prothrombotic fibrin clot properties [197, 198]. The COVID‐19 pandemic further exemplified this context‐dependence [25, 199]. Across chronic stable CAD, systemic inflammatory cardiovascular injury, and acute coronary thrombosis, the same circulating molecule may carry different biological significance. This context‐dependence limits the applicability of any single marker or threshold across disease states.

### 6.2 NET Signatures in Human Atherosclerotic and Myocardial Tissues

NETs have been directly identified within human atherosclerotic and thrombotic tissues. Megens et al. detected NET structures at the luminal surface of human atherosclerotic plaques using immunofluorescence [164], and de Boer et al. found neutrophils and NETs within coronary thrombi from AMI patients [111]. A systematic histopathological analysis by Pertiwi et al. extended these observations, showing that NETs were present across all subtypes of thrombotic and hemorrhagic complications of coronary AS, including plaque rupture, erosion, and intraplaque hemorrhage [180]. The consistent identification of NETs in these distinct pathological categories indicates that NET involvement is a shared feature of coronary thrombo‐inflammation rather than a mechanism‐specific phenomenon.

These histological observations have been complemented by mechanistic work, although the line between human clinical findings and in vitro extensions should be drawn clearly. A human in vivo observation measured NET markers and MCP‐1 levels in culprit‐site blood from STEMI patients and found both to be elevated relative to peripheral samples [200]. An in vitro experiment suggested that stimulation of human coronary artery endothelial cells with isolated NETs induced MCP‐1 and intercellular adhesion molecule 1 expression. MCP‐1, in turn, promoted further NET formation and directed monocyte migration toward fibrocyte transdifferentiation. This positive feedback loop is biologically coherent, but the controlled culture conditions may not recapitulate the hemodynamic and cellular complexity of the coronary thrombus microenvironment. Similarly, a clinical study observed that fibrocyte accumulation at infarcted sites in STEMI patients correlated with local NET burden and left ventricular dysfunction [185], whereas the causal link between NETs and fibroblast activation was established in cell culture. Likewise, He et al. observed an association between NET activity and ventricular aneurysm formation after ischemic injury in patients, with in vitro data implicating Smad, mitogen‐activated protein kinase, and RhoA signaling in NET‐driven fibroblast migration [201]. In each case, the human tissue observation is correlative, and the mechanistic chain derives from ex vivo or in vitro models that await confirmation by interventional studies.

Recent applications of spatial and single‐cell transcriptomics are beginning to map the cellular neighborhoods within human atherosclerotic plaques [202, 203]. An important caveat is that these technologies primarily characterize the inflammatory cellular niches in which NETs are likely to form and act. They do not directly quantify NET structures, histone citrullination, or extracellular DNA scaffolds. Nonetheless, they define the spatial context of neutrophil activation within vulnerable plaques. In doing so, they provide an essential framework for future studies that interrogate NETosis and its functional consequences at single‐cell resolution.

### 6.3 Translational Potential, Implementation Barriers, and Future Directions

The translational appeal of NET‐related markers lies in their potential to identify patients with heightened thrombo‐inflammatory risk who might benefit from targeted anti‐inflammatory or anti‐NET therapies. Realizing this potential, however, requires crossing several barriers that the field has not yet addressed.

In the setting of chronic plaque progression, Langseth et al. reported that circulating NET markers were associated with adverse outcomes in patients with stable CAD [204]. They proposed that persistent NET activity may reflect ongoing subclinical plaque rupture and repair. This hypothesis is biologically plausible, but it rests on small studies without independent replication. Moreover, low‐grade chronic NETosis has not been distinguished from other causes of persistent neutrophil activation in stable CAD. A systematic review of neutrophil‐enriched biomarkers in acute coronary syndrome found that elevated MPO and NGAL were each associated with higher MACE rates [205], with MPO showing independent predictive value in most included studies. Elevated dsDNA levels were also associated with worse clinical outcomes [193, 206]. However, heterogeneity in assay platforms, sample matrices, endpoint definitions, and follow‐up durations across studies makes it difficult to derive generalizable thresholds or compare effect sizes.

Genetic evidence offers a complementary perspective on NET clearance as a risk modifier. Hofbauer et al. found that the DNase 1 Q222R polymorphism, which reduces DNase 1 enzymatic activity, was independently associated with cardiovascular and all‐cause mortality in 711 STEMI patients [207]. Homozygous carriers had lower DNase activity and a higher dsDNA‐to‐DNase ratio. This shifts the conceptual focus from NET formation alone to the balance between formation and clearance. It also raises the theoretical possibility of using genetic markers of NET degradation capacity for patient stratification.

Several unresolved issues stand between current evidence and clinical implementation. First, assay standardization is lacking across the field. CitH3, the most NET‐specific circulating marker, undergoes rapid proteolytic degradation in plasma, and validated quantitative assays have only recently been developed [79]. Second, preanalytical variability poses a further challenge. The concentrations of cfDNA and dsDNA are sensitive to the choice of sample matrix (serum versus plasma), anticoagulant type, processing delay, and freeze–thaw cycles. Without rigorous control of these variables, results remain difficult to compare across studies, and reference ranges cannot be established. Third, biological specificity is limited for the most commonly measured markers, as discussed above. Perhaps the most fundamental barrier is clinical actionability. Even if a NET marker reliably identifies high‐risk patients, it remains unclear whether this information would change therapeutic decisions. No prospective trial has shown that NET marker‐guided therapy selection improves cardiovascular outcomes relative to standard care. Until such evidence emerges, the rationale for routine clinical testing is insufficient. One promising direction is to integrate NET‐specific markers with coronary imaging data, such as plaque morphology from intravascular imaging or computed tomographic angiography (CTA). This combination may improve identification of the patients most likely to benefit from anti‐inflammatory or anti‐NET interventions. Achieving this will require prospective, multicenter studies with standardized preanalytical protocols, prespecified analytical plans, and clinically meaningful endpoints. Until such studies are completed, NET‐related markers remain informative research tools for dissecting thrombo‐inflammatory biology rather than established components of clinical cardiovascular risk assessment.

## 7 Therapeutic Targeting of NETs in CVD

NETs have been increasingly recognized as central mediators of vascular injury in CVD. As discussed in preceding sections, NETs drive endothelial dysfunction, amplify thrombo‐inflammation, and destabilize atherosclerotic plaques through coordinated actions of their structural and enzymatic components. This mechanistic understanding has prompted the development of strategies that target distinct stages of NET‐mediated pathology. Current approaches fall into four broad categories: inhibition of pathological NET formation, degradation and clearance of existing NET structures, modulation of NET‐driven downstream signaling, and precision strategies tailored to specific disease stages (Tables 1 and 2). A growing number of these interventions have advanced from preclinical proof‐of‐concept to clinical evaluation, although significant translational challenges remain.

**TABLE 1:** Preclinical and clinical strategies targeting NETs in cardiovascular disease.

Biological targets	Drugs/method	Diseases/models	Mechanism/effect	Study	References
**Targeting NET formation**					
Formation	Secretory leukocyte protease inhibitor (SLPI)	–(in vitro)	Inhibits neutrophil elastase‐mediated NET release	Preclinical	[208]
Formation	Gallic acid	–(in vitro)	Attenuates free radical formation and suppresses apoptosis‐associated NET release	Preclinical	[209]
Formation	Fibrin	Sterile occlusive thrombi	Suppresses shear‐induced NET generation	Preclinical	[210]
Formation	Neonatal NET‐inhibitory factor (nNIF)	Ischemic stroke	Enhances motor performance and inhibits neuronal apoptosis	Preclinical	[211, 212]
JAK2/STAT3	Ruxolitinib	Ischemic stroke	Suppresses LPS‐induced NLRP3 inflammasome activation via JAK2/STAT3 pathway inhibition	Preclinical	[213]
Formation	Simvastatin	Cerebral ischemia	Reduces infarct area and suppresses NET‐associated inflammation	Preclinical	[214, 215, 216]
PAD4	Collagen IV‐targeted nanoparticles (Col IV NP) deliver GSK484	Atherosclerosis	Inhibits NET deposition in the injured intima while preserving endothelial barrier integrity	Preclinical	[217]
PAD4	GSK199	Ischemic stroke	Reduces infarct volume, improves long‐term functional recovery, and increases poststroke survival	Preclinical	[211]
PAD4	Cl‐amidine, F‐amidine	Atherosclerosis; stroke	Inhibits NET formation, decreases plaque size, and delays carotid artery thrombosis	Preclinical	[170, 218]
MPO	4‐Aminobenzoic acid hydrazide (4‐ABAH)	Atherosclerosis	Reduces inflammation, oxidative stress, and endothelial dysfunction, collectively inhibiting plaque development	Preclinical	[219]
MPO	AZM198 (2‐thioxanthine)	Atherosclerosis	Stabilizes unstable atherosclerotic plaques	Preclinical	[220]
MPO	AZD4831/mitiperstat	–(in vitro/Phase I)	Irreversible MPO inhibitor, reducing MPO‐mediated oxidative stress and vascular dysfunction		[221, 222]
ROS	Vitamin C/dehydroascorbic acid (DHA)	Ischemic stroke	Enhances postreperfusion cerebral blood flow, improving neurological outcomes and lowering mortality	Preclinical	[223, 224]
ROS	Diphenyleneiodonium (DPI)	Ischemic stroke	Reduces ischemia‐induced blood–brain barrier disruption and enhances neurological recovery	Preclinical	[225]
ROS	Edaravone dexborneol (Eda.B)	Acute ischemic stroke	Improves neurological function, increases cerebral blood flow, and reduces infarct volume	Preclinical	[226]
Cathepsin	Cathepsin S inhibitor	Atherosclerosis	Reduces elastic lamina breaks, intraplaque macrophage accumulation, and buried fibrous caps	Preclinical	[227]
**Targeting NET structure**					
DNA scaffold	DNase I	Ischemic stroke	Promotes NET lysis and enhances tPA‐mediated thrombolysis	Preclinical	[218]
DNA scaffold	DNase I	Diabetic atherosclerosis	Reduces plaque‐associated NETs and inflammation, supporting plaque regression after lipid‐lowering therapy	Preclinical	[143]
DNA scaffold	DNase I	Atherosclerosis	Mitigates vascular intimal damage in a murine model of arterial injury	Preclinical	[87, 228]
Histones	Antihistone H4 antibodies	Atherosclerosis	Inhibits histone H4‐mediated membrane lysis, promoting atherosclerotic plaque stability	Preclinical	[163]
Histones	Small polyanions (SPAs)	Cardiac ischemia–reperfusion injury	Dismantles the NET scaffold by displacing histones from the DNA backbone	Preclinical	[229]
HMGB1	Glycyrrhizin	Cerebral ischemia/reperfusion injury	Downregulates the HMGB1/TLR4/NF‐κB signaling axis, reducing neuroinflammation	Preclinical	[230, 231]
HMGB1	Berberine	Ischemic stroke	Attenuates ischemia–reperfusion injury by inhibiting HMGB1 release and NF‐κB nuclear translocation	Preclinical	[232]
DNA	DNase 1	Ischemic stroke	Enhances ex vivo dissolution of patient thrombi	Clinical	[233]
**Others**					
Neutrophil recruitment	CCR2 antagonist (RS102895)	Atherosclerosis	Diminishes myeloid cell adhesion, plaque growth, and macrophage aggregation via chrono‐pharmacological targeting	Preclinical	[234]
—	Bivalirudin	STEMI	Attenuates the postprocedural rise in NET formation	Clinical	[235]
Interleukin (IL)‐1β	Canakinumab	Cardiovascular events in patients with prior MI	Lowered the incidence of major adverse cardiovascular events	Clinical (Phase III)	[236, 237]
Interleukin‐6 receptor	Tocilizumab	ST‐segment elevation myocardial infarction (STEMI)	Increased the myocardial salvage index	Clinical (Phase II)	[238]

Abbreviations: 4‐ABAH, 4‐aminobenzoic acid hydrazide; CCR2, C–C motif chemokine receptor 2; DHA, dehydroascorbic acid; DPI, diphenyleneiodonium; Eda.B, edaravone dexborneol; HMGB1, high mobility group box 1; JAK2, Janus kinase 2; MPO, myeloperoxidase; NET, neutrophil extracellular trap; NF‐κB, nuclear factor kappa‐light‐chain‐enhancer of activated B cells; NLRP3, NLR family pyrin domain containing 3; nNIF, neonatal NET‐inhibitory factor; PAD4, peptidylarginine deiminase 4; ROS, reactive oxygen species; SLPI, secretory leukocyte protease inhibitor; SPA, small polyanion; STAT3, signal transducer and activator of transcription 3; TLR4, Toll‐like receptor 4; tPA, tissue‐type plasminogen activator.

**TABLE 2:** Clinical trials relevant to NET‐targeted therapeutic strategies in cardiovascular disease.

Trial	Drug/intervention	Target/pathway	Registry/references	Phase	Population and primary endpoint	Key findings and status
**Inhibition of NET formation**						
COLCOT	Colchicine (0.5 mg/d)	Tubulin/NETosis	NCT02551094 [239, 240]	III	Recent MI (≤30 d); *n* = 4745; primary: composite of CV death, cardiac arrest, MI, stroke, urgent revascularization	23% MACE reduction (HR 0.77; 95% CI 0.61–0.96); inhibits NET formation post‐PCI. FDA‐approved 2023; completed
LoDoCo2	Colchicine (0.5 mg/d)	Tubulin/NETosis	ACTRN 12614000093684 [241, 242]	III	Chronic coronary disease, stable ≥6 mo; *n* = 5522; primary: composite of CV death, MI, ischemic stroke, revascularization	31% MACE reduction (HR 0.69; 95% CI 0.57–0.83); benefit sustained over 5 years; completed
CLEAR	Colchicine (0.5 mg/d)	Tubulin/NETosis	NCT03048825 [243]	III	Acute STEMI + PCI; *n* = 7062; primary: composite of CV death, MI, stroke, revascularization	Neutral: no reduction in primary endpoint over 3‐year follow‐up when started acutely post‐MI; completed
SATELLITE	Mitiperstat (AZD4831, 5 mg/d)	MPO (irreversible)	NCT03756285 [244]	IIa	HFpEF/HFmrEF (LVEF ≥40%); *n* = 41; primary: MPO specific activity	>50% MPO activity reduction; 75% placebo‐adjusted; safe; terminated early (COVID‐19); completed
ENDEAVOR	Mitiperstat (AZD4831, 2.5/5 mg/d)	MPO (irreversible)	NCT04986202 [245]	IIb/III	HFpEF/HFmrEF (LVEF >40%); ∼1480; primary: Δ6MWD and ΔKCCQ‐TSS at 16 week	Sequential IIb–III design testing MPO inhibition on exercise capacity and symptoms; completed
**Degradation and clearance of NETs**						
NETs‐target	Dornase alfa (rhDNase I)	NET DNA scaffold	NCT04785066	II	Acute ischemic stroke postthrombectomy; primary: arterial recanalization	Evaluating dornase alfa for postthrombectomy recanalization in large‐vessel stroke; completed
EXTEND‐IA DNase	Dornase alfa (IV)	NET DNA scaffold	NCT05203224	II	Acute large‐vessel ischemic stroke (adjunct to thrombolysis/thrombectomy); Bayesian dose‐finding	Adaptive dose‐finding trial (Australia) assessing IV dornase alfa as reperfusion adjunct; recruiting
ReScinD	Dornase alfa (IV, 500 µg/kg)	cfDNA/AIM2 inflammasome	NCT05880524	II	Acute ischemic stroke (≤24 ± 6 h); primary: systemic immune activation reduction	Testing whether cfDNA neutralization prevents AIM2‐driven recurrent plaque destabilization; unknown status
**Targeting NET‐driven downstream signaling**						
CANTOS	Canakinumab (150 mg SC q3m)	IL‐1β/NLRP3	NCT01327846 [237]	III	Prior MI + hsCRP ≥2 mg/L; *n* = 10,061; primary: nonfatal MI, nonfatal stroke, CV death	15% MACE reduction (HR 0.85; 95% CI 0.74–0.98); residual IL‐18/IL‐6 in some patients; completed
ASSAIL‐MI	Tocilizumab (single IV, 280 mg)	IL‐6 receptor	NCT03004703 [238]	II	Acute STEMI + PCI; *n* = 199; primary: myocardial salvage index (MRI)	Increased salvage index; secondary analysis: reduced NET markers (dsDNA, MPO–DNA, CitH3); completed
RESCUE	Ziltivekimab (7.5/15/30 mg SC q4w)	IL‐6 ligand	NCT03926117 [246]	II	CKD 3–5 + hsCRP ≥2 mg/L; *n* = 264; primary: % change in hsCRP at 12 week	Dose‐dependent hsCRP reduction (up to 92%); reduced fibrinogen and SAA; completed
ZEUS	Ziltivekimab (15 mg SC monthly)	IL‐6 ligand	NCT05021835 [247]	III	ASCVD + CKD + hsCRP ≥2 mg/L; *n* = 6376; primary: composite MACE	Multinational Phase III testing IL‐6 inhibition on MACE in high‐risk residual inflammation; recruiting
**Anticoagulant‐based NET modulation**						
COMPASS	Rivaroxaban (2.5 mg bid) + aspirin	FXa/NET–FX loop	NCT01776424 [248]	III	Stable CAD or PAD; *n* = 27,395; primary: CV death, MI, stroke	24% MACE reduction (HR 0.76; 95% CI 0.66–0.86); mechanistic link to NET–FX disruption; completed

Abbreviations: 6MWD, 6‐min walk distance; AIM2, absent in melanoma 2; ASCVD, atherosclerotic cardiovascular disease; CAD, coronary artery disease; cfDNA, cell‐free DNA; CitH3, citrullinated histone H3; CKD, chronic kidney disease; CV, cardiovascular; dsDNA, double‐stranded DNA; FXa, activated factor X; HFmrEF, heart failure with mildly reduced ejection fraction; HFpEF, heart failure with preserved ejection fraction; hsCRP, high‐sensitivity C‐reactive protein; KCCQ‐TSS, Kansas City Cardiomyopathy Questionnaire total symptom score; LVEF, left ventricular ejection fraction; MACE, major adverse cardiovascular events; MI, myocardial infarction; MPO, myeloperoxidase; NET, neutrophil extracellular trap; NLRP3, NLR family pyrin domain containing 3; PAD, peripheral artery disease; PCI, percutaneous coronary intervention; SAA, serum amyloid A; SC, subcutaneous; STEMI, ST‐elevation myocardial infarction.

*Data sources*: trial registration details from ClinicalTrials.gov (https://clinicaltrials.gov) and, for LoDoCo2, the Australian New Zealand Clinical Trials Registry (ANZCTR, https://www.anzctr.org.au; ACTRN12614000093684); reported findings from the cited publications.

### 7.1 Inhibition of Pathological NET Formation

Preventing excessive NET formation at its source is the most extensively studied therapeutic strategy. Multiple molecular checkpoints in the NET formation cascade offer potential intervention targets, including PAD4‐mediated chromatin decondensation, NADPH oxidase‐dependent ROS generation, and granular protease activation.

Given the central role of PAD4 in NET chromatin remodeling, pharmacological inhibition of PAD4 by Cl‐amidine and F‐amidine reduced plaque burden, delayed carotid thrombosis, and improved stroke outcomes [170, 218]. Targeted delivery approaches have further improved the therapeutic index of PAD4 inhibitors. In murine AS models, Collagen IV‐directed nanoparticles loaded with GSK484 selectively inhibited NET deposition at sites of intimal injury while preserving endothelial barrier function [217]. This nanoparticle‐based strategy may enhance local drug concentrations at vulnerable lesion sites and limit off‐target immunosuppression, although its scalability and clinical feasibility await further evaluation.

Oxidative stress is another important trigger for NET release. Vitamin C and its blood–brain barrier‐permeable derivative, dehydroascorbic acid, improved reperfusion cerebral blood flow and reduced mortality in experimental stroke models [223, 224, 249, 250]. Diphenyleneiodonium, an NADPH oxidase inhibitor, attenuated blood–brain barrier disruption, while edaravone dexborneol reduced infarct volume and improved neurological function in ischemic settings [225, 226]. These results suggest that ROS scavenging may indirectly limit NET formation during ischemic cardiovascular events, although the specificity of such interventions for NET‐related pathology has not been fully established.

Agents targeting granular proteases have also shown anti‐NET activity. Secretory leukocyte protease inhibitors suppress NETosis through inhibition of NE, and gallic acid reduces NET formation by attenuating free radical production [208, 209]. Several anti‐inflammatory compounds inhibit NET release through distinct mechanisms. The JAK2 inhibitor ruxolitinib suppressed NLRP3 inflammasome activation and reduced ischemic injury in preclinical stroke models [213]. Simvastatin, widely used for lipid‐lowering in CVD patients, decreased both infarct size and NET‐associated inflammation in experimental cerebral ischemia [216]. Of note, a pilot randomized controlled clinical trial demonstrated that high‐dose simvastatin improved neutrophil function and clinical outcomes in patients with pneumonia and sepsis [216]. This finding suggests that statins may exert clinically relevant anti‐NET effects beyond lipid modulation. More recently, neonatal NET‐inhibitory factors (nNIF) have been shown to suppress NET formation and improve neuronal survival and functional recovery after stroke in animal models [211, 212].

MPO is another attractive therapeutic target, given its dual role in NET formation and in generating cytotoxic reactive species that amplify vascular injury. First‐generation inhibitors such as 4‐ABAH reduced inflammation and plaque development in atherosclerotic mice [219]. The more selective irreversible MPO inhibitor AZM198 stabilized pre‐existing unstable atherosclerotic plaques in preclinical studies [220, 221, 222]. Importantly, the first‐in‐class clinical MPO inhibitor AZD4831 (mitiperstat) has completed a phase IIa trial (SATELLITE, NCT03756285) in patients with heart failure with preserved ejection fraction [244]. In that trial, it achieved over 50% reduction in MPO activity with an acceptable safety profile. A sequential Phase IIb/III trial (ENDEAVOR) is currently evaluating whether mitiperstat improves exercise capacity and symptoms in these patients [245]. These advances illustrate the clinical feasibility of targeting NET‐related enzymes in cardiovascular populations.

Several existing cardiovascular therapies also exhibit anti‐NET properties. Because platelet activation is required for platelet‐mediated NET release, aspirin can indirectly suppress NET formation and thereby slow AS progression. The anticoagulant heparin disrupts NET scaffolds with efficiency comparable to DNase I, raising the possibility that conventional anticoagulants may confer cardiovascular benefit partly through NET‐related mechanisms. Consistent with this, the COMPASS trial demonstrated that adding low‐dose rivaroxaban to aspirin reduced atherosclerotic events, cardiovascular mortality, and all‐cause death in patients with stable CAD [251]. Subsequent mechanistic analyses revealed that the Gla domain of factor X mediates its binding to activated neutrophils, promoting the formation of NET–FX complexes with enhanced procoagulant activity [252, 253, 254]. These findings suggest that rivaroxaban may protect against cardiovascular events not only through direct anticoagulation but also by disrupting the positive feedback loop between factor X and NETs.

### 7.2 Degradation and Clearance of Excessive NETs

Once formed, NETs propagate vascular injury through their DNA scaffolds and associated cytotoxic proteins. Strategies to dismantle these structures or neutralize their bioactive components therefore represent a complementary therapeutic approach.

Exogenous DNase I effectively degrades extracellular DNA and promotes thrombus resolution in preclinical models of arterial injury, diabetes, and ischemic stroke [143, 218], and DNase I‐based strategies confer significant cardioprotection [182]. However, native DNase I has low serum stability and is susceptible to inactivation by G‐actin, which limits its therapeutic utility. To address this, dual‐active DNase1 variants with enhanced activity against both free DNA and chromatin‐bound DNA have been engineered [228]. These modified enzymes represent a promising next‐generation approach for NET clearance, although their in vivo pharmacokinetics require further optimization [255]. Clinical translation of DNase‐based therapy is actively underway. Early studies showed that DNase enhances ex vivo thrombus dissolution in stroke patients [233]. Recombinant human DNase (dornase alfa), already FDA‐approved for cystic fibrosis, is currently being evaluated in ischemic stroke across multiple clinical trials. The EXTEND‐IA DNase trial (NCT05203224), a Bayesian dose‐finding Phase II study in Australia, is assessing intravenous dornase alfa as an adjunct to thrombolysis and thrombectomy in acute large‐vessel ischemic stroke (NCT05203224). A parallel Phase II trial (NETs‐target, NCT04785066) in France is evaluating dornase alfa for arterial recanalization in postthrombectomy patients. A separate randomized placebo‐controlled Phase II trial (NCT05880524) at Ludwig Maximilian University of Munich is testing whether intravenous DNase I reduces systemic immune activation after acute ischemic stroke. It should be noted that DNase‐based approaches primarily target the DNA scaffold and may not fully neutralize the proteinaceous effectors of NETs, such as histones and granular enzymes. This likely explains the variable efficacy of DNase across different disease models. In experimental aortic aneurysm, for instance, DNase failed to confer therapeutic benefit despite effective NET disruption, whereas Cl‐amidine treatment was protective [256]. These results suggest that in that context the protein components rather than the DNA scaffold itself serve as the principal effectors of pathology in that particular context. The choice between DNA‐targeted and protein‐targeted strategies may therefore depend on the dominant pathological mechanism at each disease stage.

Histones are the most abundant proteins associated with NET structures and exert potent cytotoxic and prothrombotic activity. Neutralization with antihistone antibodies stabilized atherosclerotic plaques in murine models [163]. Small polyanions displaced histones from NET scaffolds and alleviated cardiac ischemia–reperfusion injury [229]. More recently, cyclic histone interference peptides have been shown to limit lesional cell death and reduce myeloid cell recruitment to atherosclerotic plaques [257]. These histone‐targeted strategies may neutralize a major cytotoxic effector without completely dismantling the NET structure, potentially preserving residual antimicrobial function.

Other NET‐associated alarmins contribute to inflammatory amplification. HMGB1 released from NETs activates TLR4/NF‐κB signaling and sustains a cycle of neutrophil recruitment and tissue injury. Glycyrrhizin and berberine have been shown to attenuate this pathway, reducing neuronal apoptosis and inflammation in cerebral ischemia–reperfusion models [231, 232]. While promising as proof‐of‐concept, the clinical applicability of these natural compounds for NET‐related cardiovascular conditions remains to be determined.

### 7.3 Targeting NET‐Driven Downstream Signaling

Rather than targeting NETs directly, modulation of the downstream inflammatory and thrombotic cascades triggered by NETs represents a parallel therapeutic strategy. This approach may have broader applicability, as many NET‐driven pathways converge on common inflammatory mediators that also contribute to AS through NET‐independent mechanisms.

NETs recruit myeloid cells and amplify cytokine‐driven inflammation, thereby accelerating vascular injury [234]. Pharmacological inhibition of CCR2 signaling reduced monocyte adhesion, macrophage infiltration, and plaque growth in atherosclerotic mice, demonstrating that chemokine modulation can attenuate NET‐mediated inflammatory amplification [234]. Large‐scale clinical trials have provided proof‐of‐concept that targeting downstream inflammatory pathways improves cardiovascular outcomes. In the CANTOS trial, IL‐1β inhibition with canakinumab reduced MACE in patients with prior myocardial infarction and residual inflammatory risk [236, 237]. In the ASSAIL‐MI trial, IL‐6 receptor blockade with tocilizumab increased myocardial salvage in patients with STEMI [238]. Notably, a secondary analysis of this trial revealed that tocilizumab also reduced circulating NET markers, including dsDNA, MPO–DNA complexes, and citrullinated histone H3, providing a direct link between IL‐6 signaling and NET biology. The IL‐6 ligand itself is now being targeted. In the Phase II RESCUE trial, ziltivekimab, a monoclonal antibody targeting the IL‐6 ligand, produced marked reductions in inflammatory biomarkers in the phase II RESCUE trial among patients with chronic kidney disease (CKD) and elevated hsCRP [246]. Building on this, the ongoing ZEUS trial (NCT05021835), a multinational Phase III study enrolling 6376 participants with atherosclerotic CVD, CKD, and systemic inflammation, is now testing whether ziltivekimab reduces MACE [247]. If successful, ZEUS would provide a fully novel approach for secondary cardiovascular prevention in patients with CKD. Single‐cytokine blockade may nonetheless be incomplete. A CANTOS subanalysis showed that some patients retained residual inflammatory activation despite effective IL‐1β blockade, with persistent elevations in IL‐18 and IL‐6. This observation raises the possibility that NLRP3 inflammasome inhibition or combined IL‐1β/IL‐18 blockade may offer more complete anti‐inflammatory coverage than targeting a single cytokine alone [258].

Colchicine represents the most clinically advanced anti‐NET strategy. It suppressed NET formation in ACS patients undergoing PCI [240], and three landmark trials have defined its clinical profile (Table 2). COLCOT and LoDoCo2 demonstrated significant MACE reductions in post‐MI and chronic coronary disease settings, respectively [239, 241, 259], leading to FDA approval for secondary cardiovascular prevention. A meta‐analysis of five randomized trials (>11,000 patients) confirmed a 22% MACE reduction with long‐term low‐dose colchicine [258]. However, the CLEAR trial and a subsequent large‐scale randomized study reported neutral results when colchicine was initiated acutely after STEMI [243, 260], suggesting that the timing of initiation relative to the ischemic event critically influences efficacy. A recent preclinical study showed that colchicine inhibited NETs and attenuated cardiac remodeling in a murine acute MI model by reducing NOX2/ROS production and Ca^2^^+^ influx [261]. The mechanistic rationale therefore remains valid despite inconsistent acute‐phase clinical results. Other clinically tested agents include bivalirudin, which was associated with reduced NET release during PCI in patients with STEMI [235].

### 7.4 Precision and Stage‐Specific Intervention Strategies

A critical challenge in translating NET‐targeted therapies to clinical practice is matching the intervention to the disease stage. As discussed in Section 5, the dominant pathogenic role of NETs shifts across the atherosclerotic continuum, and their potential reparative contribution in the postinfarction setting further complicates therapeutic design. These considerations call for a calibrated, disease‐stage‐aware approach rather than uniform NET suppression. Several considerations inform the design of precision NET‐targeted interventions. First, the choice between formation inhibitors, scaffold degraders, and downstream signaling modulators should be guided by the dominant pathological mechanism at each disease stage. During stable AS, formation inhibitors (Sections 7.1 and 7.3) may be most appropriate [204]. During acute thrombotic events, when preformed NETs serve as scaffolds for thrombus expansion, DNase‐based degradation strategies may be more effective. In the postinfarction phase, targeting specific NET‐derived mediators, such as histones or HMGB1, may allow clinicians to limit tissue damage while preserving the reparative functions of neutrophils. As noted in Section 2.3, vascular NETs can persist for prolonged periods and undergo self‐renewal [44], adding further complexity to the temporal considerations in NET‐targeted therapy. Second, the safety of sustained NET inhibition requires careful evaluation, particularly given the antimicrobial functions of NETs. The key evidence and translational considerations regarding infection risk, species‐specific differences, and delivery selectivity are discussed in Section 8.2. Third, patient stratification based on NET biomarkers may improve therapeutic precision. As discussed in Section 6, circulating NET‐related markers have been associated with disease severity and prognosis in both stable CAD and ACS [204]. These biomarkers could potentially guide treatment decisions and enable treatment response monitoring, although the standardization and validation challenges discussed in Section 6.3 must first be addressed. Finally, the integration of emerging technologies is expected to refine our understanding of NET heterogeneity in cardiovascular tissues. Recent advances in single‐cell transcriptomics, spatial proteomics, and multiomics profiling have begun to reveal disease stage‐ and tissue‐specific NET signatures. For example, DNA‐sensing inflammasomes activated by NET‐derived DNA have been implicated in recurrent atherosclerotic plaque disruptions and cerebrovascular events [121]. These findings open new avenues for more selective interventions. Combining nanoparticle‐based targeted delivery, biomarker‐guided patient selection, and temporally defined treatment windows holds promise for transforming NET‐directed therapy from a broad anti‐inflammatory strategy into a precision medicine approach for CVD.

In summary, the therapeutic targeting of NETs in CVD has progressed from early preclinical studies to clinical evaluation. Multiple strategies are being actively explored, from formation inhibition to scaffold degradation to downstream signaling modulation. The dual nature of NETs as both pathological mediators and immune defenders, however, requires a careful, context‐aware approach. Future clinical studies that incorporate stage‐specific treatment algorithms, biomarker‐guided patient stratification, and targeted delivery systems will be essential to realize the full translational potential of NET‐directed cardiovascular therapy.

## 8 Knowledge Gaps, Challenges, and Future Perspectives

**FIGURE 6:**
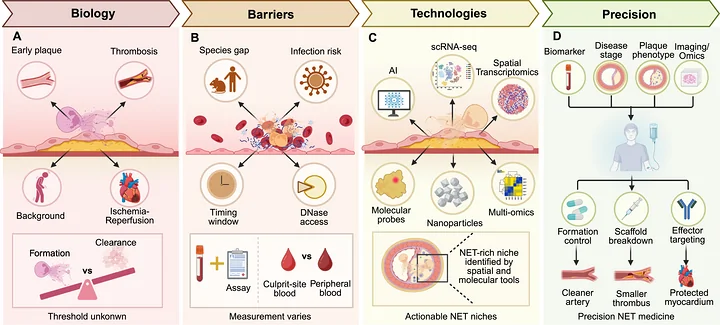
Framework for NET‐targeted therapy in atherosclerotic cardiovascular disease. (A) Context‐dependent NET biology governs the lifecycle of NETs, from formation in early plaques to clearance during thrombosis and post‐MI remodeling, all modulated by patient‐specific factors. (B) Translational hurdles and safety concerns arise from species differences, infection risks, and variable DNase responses; notably, NET measurements are highly sensitive to the sampling site. (C) Modern systems‐level approaches, including AI‐assisted multiomics and molecular probes, are essential to map NET‐associated niches. (D) The future of precision medicine lies in integrating biomarkers and multimodal imaging to tailor interventions, such as modulating NET formation or targeting specific effectors, to alleviate the thrombo‐inflammatory burden. *Created with BioRender.com. AI, artificial intelligence; DNase, deoxyribonuclease; MI, myocardial infarction; NETs, neutrophil extracellular traps.

Although NETs have emerged as active participants in AS and CVD, many fundamental questions remain unanswered. Below, we discuss the major knowledge gaps, translational hurdles, and emerging technologies that may reshape the field (Figure 6).

### 8.1 Context‐Dependent Roles of NETs in Cardiovascular Biology

As established in preceding sections, the biological effects of NETs vary across vascular beds, disease phases, and patient populations [164]. Yet most studies have examined NET activity at a single time point or in a single vascular territory. How the balance between NET formation and clearance shifts across the atherosclerotic continuum is not well understood, and longitudinal analyses are needed to define the thresholds at which NETs transition from protective to injurious. As detailed in Section 4, NET components form an integrated pathological network whose combined vascular impact exceeds the sum of individual effects. The relative contribution of each component likely varies across the disease course, but whether specific NET molecular profiles, for example, histone‐rich versus protease‐rich, correspond to distinct clinical outcomes has not been systematically studied. aggNETs can paradoxically resolve rather than amplify inflammation [262], but the molecular rules governing this functional switch are not yet defined and will require direct comparisons of NET composition and density across different cardiovascular contexts. Substantial heterogeneity also exists among patients. Metabolic comorbidities modulate the neutrophil phenotype. Neutrophils from diabetic patients exhibit elevated PAD4 expression and increased susceptibility to NETosis, which may partly explain the accelerated AS observed in this population [263]. Aging similarly enhances NET‐forming capacity while impairing clearance [7]. Concurrent infection, as highlighted during the COVID‐19 pandemic, can amplify NET‐driven immunothrombosis in patients with underlying AS [264]. Quantifiable indicators of individual NET status, such as neutrophil surface markers including CD177 and CD66b, circulating PAD4 levels, and plasma NET remnant concentrations, may help stratify patients according to NET‐related cardiovascular risk. Establishing and validating such panels should be a priority for future clinical studies.

### 8.2 Translational Hurdles and Safety Considerations

Translating NET‐targeted therapies from bench to bedside faces several interconnected challenges. Most preclinical evidence comes from murine models with genetic deficiencies or pharmacological interventions such as Cl‐amidine and DNase I. Murine neutrophils differ from human neutrophils in lifespan, circulating counts, and NET structural composition. PAD4 deficiency in hematopoietic cells did not significantly alter plaque size in hypercholesterolemic mice despite reducing arterial thrombosis [87], raising questions about how faithfully these models predict human responses [265]. Safety is another concern. NETs serve essential roles in antimicrobial defense, and prolonged PAD4 blockade or global NET degradation with DNase I may increase infection susceptibility. PAD4‐deficient mice do not exhibit marked immunodeficiency under standard housing conditions [266], but this finding may not translate to chronically ill patients who face frequent pathogen exposure. Colchicine, which indirectly suppresses NETosis, offers a real‐world reference. In the COLCOT trial, a small numerical increase in pneumonia incidence was observed [239]. Long‐term safety data specifically addressing infection risk with NET‐targeted agents remain essential before these therapies enter routine clinical use. The variable performance of DNase I across disease models reflects the physical complexity of mature NETs. As noted in Section 2.3, the oxidative plaque environment impairs enzymatic degradation. Moreover, when integrated into thrombi or embedded within plaques, NET scaffolds are further shielded by histones and granule proteins, limiting enzymatic access [267]. Engineered DNase variants with enhanced activity, or combination strategies that target both the DNA backbone and cytotoxic NET‐bound proteins, may offer improved efficacy [228]. The therapeutic window for NET intervention also needs clarification. During AMI, NETs accumulate rapidly within coronary thrombi and contribute to microvascular obstruction, suggesting that early degradation may be beneficial. However, during the resolution phase of ischemia–reperfusion injury, premature NET clearance might disrupt reparative macrophage polarization and impair healing. This temporal complexity implies that treatment should be matched to the disease phase. In the acute setting, rapid‐acting interventions such as DNase I or antihistone antibodies could reduce thrombus burden. For chronic AS, sustained low‐intensity approaches such as low‐dose colchicine or selective PAD4 inhibitors may be better suited. Future trials should incorporate phase‐specific endpoints and serial NET biomarker monitoring.

### 8.3 Emerging Technologies and Systems‐Level Approaches

Recent technological advances offer new opportunities to address these gaps. Single‐cell RNA sequencing has revealed previously unrecognized neutrophil heterogeneity within atherosclerotic plaques, identifying subpopulations with distinct transcriptional programs [268]. A recently published integrated single‐cell atlas of human plaques, covering approximately 250,000 cells, identified plaque‐specific neutrophil markers that were undetectable in individual datasets [202]. Spatial transcriptomics extends these findings by mapping NET‐producing neutrophils within different plaque regions and showing how they interact with adjacent endothelial cells, macrophages, and smooth muscle cells in situ [203]. These spatially resolved datasets will be important for identifying the cellular neighborhoods most vulnerable to NET‐mediated injury.

Combining proteomic, metabolomic, and epigenomic profiling may reveal how NET molecular composition shifts across the atherosclerotic continuum and identify NET signatures associated with specific outcomes. Citrullinomics, the systematic profiling of citrullinated substrates, could uncover PAD4‐dependent pathways beyond chromatin decondensation that contribute to vascular pathology [269]. The intersection of NET biology with clonal hematopoiesis of indeterminate potential (CHIP) is also gaining attention. CHIP‐associated mutations in TET2 and DNMT3A enhance neutrophil inflammatory responses and may promote excessive NETosis [167]. Determining whether CHIP carriers exhibit heightened NET‐driven vascular injury could help refine individual risk assessment and guide treatment decisions.

Machine learning and artificial intelligence are increasingly applied to cardiovascular research and may accelerate progress in the NET field. Deep learning algorithms can integrate multiomics datasets to identify NET‐related molecular signatures that predict plaque vulnerability or adverse cardiovascular events [8]. AI‐assisted image analysis could automate the quantification of NETs in histological sections and spatial transcriptomics data, reducing operator‐dependent variability and enabling large‐scale studies. Computational drug screening platforms may also help identify selective PAD4 inhibitors or novel NET‐modulating compounds with favorable safety profiles [270]. As NET biology generates increasingly high‐dimensional data, these computational tools will become important for extracting clinically actionable insights.

Molecular probes for NET‐specific epitopes, including citrullinated histone H3 and MPO–DNA complexes, could enable noninvasive monitoring of NET burden in patients. They would also help evaluate therapeutic responses in clinical trials. Such probes are not yet available for clinical use, and their development represents an active area of research. Nanoparticle‐based drug delivery offers a complementary strategy. Targeted nanoparticles directed against Collagen IV exposed at erosion sites or against activated endothelial markers can deliver PAD4 inhibitors or DNase I selectively to diseased vascular segments [217]. This selectivity improves precision while minimizing off‐target effects. Challenges in bioavailability, hepatic clearance, and long‐term safety persist [18], but the concept holds considerable promise.

The future of NET‐targeted therapy lies in precision, phase‐specific interventions rather than a uniform approach. For patients with chronic stable AS and residual inflammatory risk despite optimized lipid‐lowering therapy, low‐dose anti‐inflammatory agents with indirect anti‐NET properties may be the most appropriate option. The clinical experience with colchicine validates the principle that anti‐inflammatory strategies with anti‐NET activity can reduce cardiovascular events in chronic coronary disease [239, 240, 241]. However, the neutral results in acute STEMI underscore the need for stage‐specific approaches; dedicated NET‐degrading agents with rapid onset of action, such as dornase alfa, warrant evaluation in the acute setting [233, 260]. As discussed in Section 6, the validation of circulating NET biomarkers for clinical decision‐making remains limited by assay standardization challenges and the absence of prospective companion diagnostic trials.

Together, these technological advances, from single‐cell atlases and spatial omics to AI‐driven analytics and targeted nanomedicine, are poised to enable more precise and individualized approaches to NET‐directed therapy. How these tools can be combined with stage‐specific treatment algorithms and biomarker‐guided patient stratification is addressed in the concluding section.

### 8.4 NETs as Targets in Precision Cardiovascular Medicine

The future of NET‐targeted therapy lies in precision, phase‐specific interventions. For patients with chronic stable atherosclerotic disease who retain residual inflammatory risk despite optimized lipid‐lowering therapy, low‐dose anti‐inflammatory agents with indirect anti‐NET activity may currently be the most practical option. In COLCOT, colchicine 0.5 mg daily reduced ischemic cardiovascular events after recent myocardial infarction [239], and in LoDoCo2, the same regimen reduced cardiovascular events in chronic coronary disease [271]. The 2023 FDA approval of low‐dose colchicine for secondary cardiovascular prevention was a milestone in anti‐inflammatory cardiology. Whether these benefits are partly mediated through NET suppression is an open question. Colchicine inhibits NET formation in patients with acute coronary syndrome [240], but its contribution relative to other anti‐inflammatory mechanisms has not been isolated. For patients presenting with acute coronary syndromes, more direct NET‐targeted approaches may be needed. Dornase alfa, a recombinant human DNase I currently under Phase II evaluation for ischemic stroke [272], could be repurposed for acute coronary interventions. However, the CLEAR‐Synergy trial reported neutral effects of colchicine in immediate STEMI (NCT03048825), underscoring that timing and patient selection will be critical. Future acute‐phase trials should test dedicated NET‐degrading agents with rapid onset. Biomarker‐guided selection adds a third dimension. Circulating MPO–DNA complexes, citrullinated histone H3, and cfDNA have been associated with plaque instability and adverse cardiovascular outcomes in observational studies and could in principle guide treatment decisions. However, analytical standardization of NET biomarker assays across laboratories is lacking, and no NET biomarker has been tested as a companion diagnostic in a randomized trial. Prospective validation in large, diverse cohorts remains an essential step before biomarker‐guided NET intervention becomes feasible.

Over the past two decades, research has established NETs as central mediators in the pathophysiology of AS and CVD. At the basic science level, the cooperative actions of NET components converge to promote endothelial injury, thrombo‐inflammation, and plaque instability. At the translational level, species differences, immune safety concerns, and the context‐dependent biology of NETs pose formidable barriers to clinical development. At the clinical level, colchicine has provided the first proof‐of‐concept that anti‐inflammatory strategies with anti‐NET activity can reduce cardiovascular events. In parallel, tools such as single‐cell atlases, spatial omics, AI‐driven analytics, and targeted nanomedicine are poised to enable more precise and individualized approaches. The path forward will require the integration of these advances. Mechanistic studies using multiomics and spatial technologies should define NET molecular signatures linked to specific disease outcomes. Translational programs should develop selective NET‐modulating agents and validate NET biomarkers in prospective cohorts. Clinical trials should adopt phase‐specific designs that match intervention intensity to disease acuity, with biomarker‐guided patient stratification. By connecting these three levels, from molecular mechanism through translational validation to clinical application, the field can move beyond broad immunomodulation toward precise targeting of pathological NET activity, and ultimately reduce the burden of atherosclerotic CVD.

## 9 Conclusions

AS remains a leading cause of cardiovascular morbidity and mortality worldwide [273]. This review has examined NET biology across the full pathological continuum of atherosclerotic CVD, from vascular homeostasis through atherogenesis and acute thrombotic events to postinfarction remodeling.

Three central themes emerge from this analysis. First, the pathogenic impact of NETs derives not from any single component but from the coordinated, scaffold‐mediated delivery of proteolytic, oxidative, immunostimulatory, and prothrombotic effectors at the vessel wall. This mode of action is structurally distinct from the diffuse mediator release by other inflammatory cells. Second, NET function is stage dependent, transitioning from inflammatory amplification to immunothrombotic scaffolding and ultimately to microvascular obstruction as disease progresses. This stage‐specificity argues against blanket NET suppression and instead calls for temporally matched therapeutic strategies. Third, NETs have a dual nature: they are protective in host defense yet pathogenic when dysregulated. This duality demands precision approaches that selectively limit pathological NET activity while preserving immune surveillance, a balance that current interventions have not yet achieved.

At the translational level, colchicine has provided the concept that anti‐inflammatory strategies with anti‐NET activity reduce cardiovascular events in chronic disease. Meanwhile, emerging therapies targeting NET scaffolds, specific components, and downstream signaling pathways are advancing through clinical evaluation. Circulating NET biomarkers show promise for risk stratification, although assay standardization and prospective validation remain essential. The integration of single‐cell and spatial omics, AI‐driven analytics, and targeted nanomedicine is poised to enable more precise, individualized approaches. Translating these advances into stage‐specific, biomarker‐guided interventions will be essential for realizing the therapeutic potential of NET modulation in cardiovascular medicine.

## Author Contributions

Ji Zhang, Fangjiaqi Wei, and Mengdi Qu contributed to writing the original draft. Yuwen Shao and Tingye Xu contributed to writing and literature collection. Darko Stojkov provided critical revisions and scientific input on NET biology. Hao Zhang, Changhong Miao, Ben Huang, and Hans‐Uwe Simon supervised the work and assisted with the review and revision of the manuscript. All authors have read and approved the final manuscript.

## Funding

This review was supported by the National Natural Science Foundation of China (No. 82472183), Shanghai Pujiang Talents Program (No. 21PJD013), and Shanghai Raising Star Program (24QA2707800).

## Ethics Statement

The authors have nothing to report.

## Conflicts of Interest

The authors declare no conflicts of interest.

## Data Availability

The authors have nothing to report.

## References

[1] G. A. Roth , G. A. Mensah , C. O. Johnson , et al., “Global Burden of Cardiovascular Diseases and Risk Factors, 1990–2019: Update from the GBD 2019 Study,” Journal of the American College of Cardiology 76, no. 25 (2020): 2982–3021. 33309175 10.1016/j.jacc.2020.11.010 PMC7755038

[2] P. Song , Z. Fang , and H. Wang , “Global and Regional Prevalence, Burden, and Risk Factors for Carotid Atherosclerosis: A Systematic Review, Meta‐analysis, and Modelling Study,” The Lancet Global Health 8, no. 5 (2020): e721–e729. 32353319 10.1016/S2214-109X(20)30117-0

[3] N. Arnold and W. Koenig , “Inflammation in Atherosclerotic Cardiovascular Disease: From Diagnosis to Treatment,” European Journal of Clinical Investigation 55, no. 7 (2025): e70020. 40055964 10.1111/eci.70020 PMC12169090

[4] A. Sarraju and S. Nissen , “Atherosclerotic Plaque Stabilization and Regression: A Review of Clinical Evidence,” Nature Reviews Cardiology 21, no. 7 (2024): 487–497. 38177454 10.1038/s41569-023-00979-8

[5] K. Grodecki , J. Geers , and J. Kwiecinski , “Phenotyping Atherosclerotic Plaque and Perivascular Adipose Tissue: Signalling Pathways and Clinical Biomarkers in Atherosclerosis,” Nature Reviews Cardiology 22, no. 6 (2025): 443–455. 39743563 10.1038/s41569-024-01110-1 PMC12092191

[6] S. Zaman , J. H. Wasfy , and V. Kapil , “The Lancet Commission on rethinking coronary artery disease: Moving From Ischaemia to Atheroma,” Lancet 405, no. 10486 (2025): 1264–1312. 40179933 10.1016/S0140-6736(25)00055-8 PMC12315672

[7] H. Wang , S. J. Kim , and Y. Lei , “Neutrophil Extracellular Traps in Homeostasis and Disease,” Signal Transduction and Targeted Therapy 9, no. 1 (2024): 235. 39300084 10.1038/s41392-024-01933-x PMC11415080

[8] S. Shetty and M. Subramanian , “Neutrophil Extracellular Traps (NETs) as Drivers of Atherosclerosis: Pathogenic Mechanisms and Therapeutic Opportunities,” Pharmacology & Therapeutics 274 (2025): 108917. 40845952 10.1016/j.pharmthera.2025.108917

[9] C. C. Baaten , M. Nagy , H. M. Spronk , H. Ten Cate , and B. L. Kietselaer , “NETosis in Cardiovascular Disease: An Opportunity for Personalized Antithrombotic Treatments?,” Arteriosclerosis, Thrombosis, and Vascular Biology 44, no. 12 (2024): 2366–2370. 39602504 10.1161/ATVBAHA.124.320150

[10] L. S. C. Lok , T. W. Dennison , K. M. Mahbubani , K. Saeb‐Parsy , E. R. Chilvers , and M. R. Clatworthy , “Phenotypically Distinct Neutrophils Patrol Uninfected human and Mouse Lymph Nodes,” PNAS 116, no. 38 (2019): 19083–19089. 31484769 10.1073/pnas.1905054116 PMC6754587

[11] M. Filippi , “Neutrophil Transendothelial Migration: Updates and New Perspectives,” Blood 133, no. 20 (2019): 2149–2158. 30898863 10.1182/blood-2018-12-844605 PMC6524565

[12] J. Lavillegrand , R. Al‐Rifai , and S. Thietart , “Alternating High‐fat Diet Enhances Atherosclerosis by Neutrophil Reprogramming,” Nature 634, no. 8033 (2024): 447–456. 39232165 10.1038/s41586-024-07693-6 PMC12019644

[13] I. Mitroulis , G. Hajishengallis , and T. Chavakis , “Bone Marrow Inflammatory Memory in Cardiometabolic Disease and Inflammatory Comorbidities,” Cardiovascular Research 119, no. 18 (2024): 2801–2812. 36655373 10.1093/cvr/cvad003 PMC10874275

[14] C. Silvestre‐Roig , Q. Braster , A. Ortega‐Gomez , and O. Soehnlein , “Neutrophils as Regulators of Cardiovascular Inflammation,” Nature Reviews Cardiology 17, no. 6 (2020): 327–340. 31996800 10.1038/s41569-019-0326-7

[15] J. M. Adrover , C. del Fresno , and G. Crainiciuc , “A Neutrophil Timer Coordinates Immune Defense and Vascular Protection,” Immunity 50, no. 2 (2019): 390–402. 30709741 10.1016/j.immuni.2019.01.002

[16] B. Engelmann and S. Massberg , “Thrombosis as an Intravascular Effector of Innate Immunity,” Nature Reviews Immunology 13, no. 1 (2013): 34–45. 10.1038/nri3345 23222502

[17] B. Lazzaretto and B. Fadeel , “Intra‐ and Extracellular Degradation of Neutrophil Extracellular Traps by Macrophages and Dendritic Cells,” Journal of Immunology 203, no. 8 (2019): 2276–2290. 10.4049/jimmunol.1800159 PMC6778307 31519860

[18] Y. Yuan , C. Sun , and X. Liu , “The Role of Neutrophil Extracellular Traps in Atherosclerosis: From the Molecular to the Clinical Level,” Journal of Inflammation Research 18 (2025): 4421–4433. 40162077 10.2147/JIR.S507330 PMC11955173

[19] Y. Ma , J. Zhang , Y. Qi , Y. Lu , Y. Dong , and D. Hu , “Neutrophil Extracellular Traps in Cardiovascular Diseases: Pathological Roles and Therapeutic Implications,” Biomolecules 15, no. 9 (2025): 1263. 41008570 10.3390/biom15091263 PMC12467555

[20] V. Brinkmann , U. Reichard , and C. Goosmann , “Neutrophil Extracellular Traps Kill Bacteria,” Science 303, no. 5663 (2004): 1532–1535. 15001782 10.1126/science.1092385

[21] C. Liang , N. Lian , and M. Li , “The Emerging Role of Neutrophil Extracellular Traps in Fungal Infection,” Frontiers in Cellular and Infection Microbiology 12 (2022): 900895. 36034717 10.3389/fcimb.2022.900895 PMC9411525

[22] Z. Chang , N. Jiang , and Y. Zhang , “The TatD‐Like DNase of Plasmodium Is a Virulence Factor and a Potential Malaria Vaccine Candidate,” Nature Communications 7 (2016): 11537. 10.1038/ncomms11537 PMC4859065 27151551

[23] G. Schönrich and M. Raftery , “Neutrophil Extracellular Traps Go Viral,” Frontiers in Immunology 7 (2016): 366. 27698656 10.3389/fimmu.2016.00366 PMC5027205

[24] B. Cortjens , O. J. de Boer , and R. de Jong , “Neutrophil Extracellular Traps Cause Airway Obstruction During respiratory Syncytial Virus Disease,” Journal of Pathology 238, no. 3 (2016): 401–411. 26468056 10.1002/path.4660

[25] H. M. Al‐Kuraishy , A. I. Al‐Gareeb , H. A. Al‐hussaniy , N. A. H. Al‐Harcan , A. Alexiou , and G. E. Batiha , “Neutrophil Extracellular Traps (NETs) and Covid‐19: A New Frontiers for Therapeutic Modality,” International Immunopharmacology 104 (2022): 108516. 35032828 10.1016/j.intimp.2021.108516 PMC8733219

[26] S. Yousefi , D. Simon , D. Stojkov , A. Karsonova , A. Karaulov , and H. Simon , “In Vivo Evidence for Extracellular DNA Trap Formation,” Cell Death & Disease 11, no. 4 (2020): 300. 32355207 10.1038/s41419-020-2497-x PMC7193637

[27] F. Apel , L. Andreeva , and L. S. Knackstedt , “The Cytosolic DNA Sensor cGAS Recognizes Neutrophil Extracellular Traps,” Science Signaling 14, no. 673 (2021): eaax7942. 33688080 10.1126/scisignal.aax7942

[28] A. Warnatsch , M. Ioannou , Q. Wang , and V. Papayannopoulos , “Inflammation. Neutrophil Extracellular Traps License Macrophages for Cytokine Production in Atherosclerosis,” Science 349, no. 6245 (2015): 316–320. 26185250 10.1126/science.aaa8064 PMC4854322

[29] Y. Döring , O. Soehnlein , and C. Weber , “Neutrophil Extracellular Traps in Atherosclerosis and Atherothrombosis,” Circulation Research 120, no. 4 (2017): 736–743. 28209798 10.1161/CIRCRESAHA.116.309692

[30] M. Jiménez‐Alcázar , C. Rangaswamy , and R. Panda , “Host DNases Prevent Vascular Occlusion by Neutrophil Extracellular Traps,” Science 358, no. 6367 (2017): 1202–1206. 29191910 10.1126/science.aam8897

[31] M. Gimbrone Jr and G. García‐Cardeña , “Endothelial Cell Dysfunction and the Pathobiology of Atherosclerosis,” Circulation Research 118, no. 4 (2016): 620–636. 26892962 10.1161/CIRCRESAHA.115.306301 PMC4762052

[32] S. Shrestha , et al., “Diabetes Primes Neutrophils for Neutrophil Extracellular Trap Formation Through Trained Immunity,” Research (Wash D C) 7 (2024): 0365. 38654733 10.34133/research.0365 PMC11037460

[33] U. K. Dhawan , P. Bhattacharya , S. Narayanan , V. Manickam , A. Aggarwal , and M. Subramanian , “Hypercholesterolemia Impairs Clearance of Neutrophil Extracellular Traps and Promotes Inflammation and Atherosclerotic Plaque Progression,” Arteriosclerosis, Thrombosis, and Vascular Biology 41, no. 10 (2021): 2598–2615. 34348488 10.1161/ATVBAHA.120.316389 PMC8454501

[34] J. Liu , P. Tao , and B. Su , “Interleukin‐33 Modulates NET Formation via an Autophagy‐dependent Manner to Promote Neutrophilic Inflammation in Cigarette Smoke‐exposure Asthma,” Journal of Hazardous Materials 487 (2025): 137257. 39842125 10.1016/j.jhazmat.2025.137257

[35] I. Tabas , “Macrophage Death and Defective Inflammation Resolution in Atherosclerosis,” Nature Reviews Immunology 10, no. 1 (2010): 36–46. 10.1038/nri2675 PMC2854623 19960040

[36] A. Doran , A. Yurdagul , and I. Tabas , “Efferocytosis in Health and Disease,” Nature Reviews Immunology 20, no. 4 (2020): 254–267. 10.1038/s41577-019-0240-6 PMC7667664 31822793

[37] L. Lei , J. Liu , Y. Li , B. Huang , and Z. Zou , “Neutrophils and Neutrophil Extracellular Traps in Diabetes Mellitus and Its Complications: Mechanisms and Therapeutic Implications,” Iscience 29, no. 5 (2026): 115585. 42023151 10.1016/j.isci.2026.115585 PMC13098529

[38] N. Sendtner , R. Seitz , N. Brandl , M. Müller , and K. Gülow , “Reactive Oxygen Species across Death Pathways: Gatekeepers of Apoptosis, Ferroptosis, Pyroptosis, Paraptosis, and beyond,” International Journal of Molecular Sciences 26, no. 20 (2025): 10240. 41155529 10.3390/ijms262010240 PMC12564638

[39] A. Hakkim , B. G. Fürnrohr , and K. Amann , “Impairment of Neutrophil Extracellular Trap Degradation Is Associated With Lupus Nephritis,” PNAS 107, no. 21 (2010): 9813–9818. 20439745 10.1073/pnas.0909927107 PMC2906830

[40] V. Sisirak , B. Sally , and V. D'Agati , “Digestion of Chromatin in Apoptotic Cell Microparticles Prevents Autoimmunity,” Cell 166, no. 1 (2016): 88–101. 27293190 10.1016/j.cell.2016.05.034 PMC5030815

[41] M. Herre , J. Cedervall , N. Mackman , and A. Olsson , “Neutrophil Extracellular Traps in the Pathology of Cancer and Other Inflammatory Diseases,” Physiological Reviews 103, no. 1 (2023): 277–312. 35951483 10.1152/physrev.00062.2021 PMC9576172

[42] J. Zhang , C. Miao , and H. Zhang , “Targeting Neutrophil Extracellular Traps in Cancer Progression and Metastasis,” Theranostics 15, no. 12 (2025): 5846–5869. 40365275 10.7150/thno.111096 PMC12068306

[43] J. J. McCord , M. Engavale , and E. Masoumzadeh , “Structural Features of Dnase1L3 Responsible for Serum Antigen Clearance,” Communications Biology 5, no. 1 (2022): 825. 35974043 10.1038/s42003-022-03755-5 PMC9381713

[44] M. Santocki , A. Such , D. Drab , G. Burczyk , and E. Kolaczkowska , “NETs Persisting in Vasculature Undergo Self‐renewal With Consequences for Subsequent Infection: A Mouse Model Study,” Blood 145, no. 18 (2025): 2070–2085. 39841013 10.1182/blood.2024026643 PMC12105727

[45] A. Yurdagul , A. C. Doran , B. Cai , G. Fredman , and I. A. Tabas , “Mechanisms and Consequences of Defective Efferocytosis in Atherosclerosis,” Frontiers in Cardiovascular Medicine 4 (2017): 86. 29379788 10.3389/fcvm.2017.00086 PMC5770804

[46] “Atherosclerosis,” Nat Rev Dis Primers 5, no. 1 (2019): 57. 31420561 10.1038/s41572-019-0116-x

[47] S. van Kesteren , et al., “Endothelial Cells Modulate Immune Cell Responses During Atherosclerosis,” Trends in Immunology (2026), in press. 10.1016/j.it.2026.03.001 PMC13506140 41925412

[48] H. Wang , H. Zhang , and Y. Wang , “Regulatory T‐cell and Neutrophil Extracellular Trap Interaction Contributes to Carcinogenesis in Non‐alcoholic Steatohepatitis,” Journal of Hepatology 75, no. 6 (2021): 1271–1283. 34363921 10.1016/j.jhep.2021.07.032 PMC12888775

[49] Y. Döring , P. Libby , and O. Soehnlein , “Neutrophil Extracellular Traps Participate in Cardiovascular Diseases: Recent Experimental and Clinical Insights,” Circulation Research 126, no. 9 (2020): 1228–1241. 32324499 10.1161/CIRCRESAHA.120.315931 PMC7185047

[50] U. K. Dhawan , T. Vartak , and H. Englert , “Macrophage DNases Limit Neutrophil Extracellular Trap‐Mediated Defective Efferocytosis in Atherosclerosis,” Circulation Research 137, no. 10 (2025): 1255–1275. 41031413 10.1161/CIRCRESAHA.125.326353 PMC12542999

[51] J. I. Borissoff , I. A. Joosen , and M. O. Versteylen , “Elevated Levels of Circulating DNA and Chromatin Are Independently Associated With Severe Coronary Atherosclerosis and a Prothrombotic state,” Arteriosclerosis, Thrombosis, and Vascular Biology 33, no. 8 (2013): 2032–2040. 23818485 10.1161/ATVBAHA.113.301627 PMC3806482

[52] M. Thakur , C. V. C. Junho , S. M. Bernhard , M. Schindewolf , H. Noels , and Y. Döring , “NETs‐Induced Thrombosis Impacts on Cardiovascular and Chronic Kidney Disease,” Circulation Research 132, no. 8 (2023): 933–949. 37053273 10.1161/CIRCRESAHA.123.321750 PMC10377271

[53] B. Yipp and P. Kubes , “NETosis: How Vital Is It?,” Blood 122, no. 16 (2013): 2784–2794. 24009232 10.1182/blood-2013-04-457671

[54] H. R. Thiam , S. L. Wong , D. D. Wagner , and C. M. Waterman , “Cellular Mechanisms of NETosis,” Annual Review of Cell and Developmental Biology 36 (2020): 191–218. 10.1146/annurev-cellbio-020520-111016 PMC8499668 32663035

[55] C. Lood , L. P. Blanco , and M. M. Purmalek , “Neutrophil Extracellular Traps Enriched in Oxidized Mitochondrial DNA Are Interferogenic and Contribute to Lupus‐Like Disease,” Nature Medicine 22, no. 2 (2016): 146–153. 10.1038/nm.4027 PMC4742415 26779811

[56] D. N. Douda , M. A. Khan , H. Grasemann , and N. Palaniyar , “SK3 channel and Mitochondrial ROS Mediate NADPH Oxidase‐independent NETosis Induced by Calcium Influx,” PNAS 112, no. 9 (2015): 2817–2822. 25730848 10.1073/pnas.1414055112 PMC4352781

[57] S. Meyers , M. Crescente , P. Verhamme , and K. Martinod , “Staphylococcus aureus and Neutrophil Extracellular Traps: The Master Manipulator Meets Its Match in Immunothrombosis,” Arterioscler Thromb Vasc Biol 42, no. 3 (2022): 261–276. 35109674 10.1161/ATVBAHA.121.316930 PMC8860219

[58] B. G. Yipp , B. Petri , and D. Salina , “Infection‐induced NETosis Is a Dynamic Process Involving Neutrophil Multitasking in Vivo,” Nature Medicine 18, no. 9 (2012): 1386–1393. 10.1038/nm.2847 PMC4529131 22922410

[59] L. Gigon , T. Fettrelet , and M. Miholic , “Syntaxin‐4 and SNAP23 Are Involved in Neutrophil Degranulation, but Not in the Release of Mitochondrial DNA During NET Formation,” Frontiers in immunology 14 (2023): 1272699. 37885878 10.3389/fimmu.2023.1272699 PMC10599146

[60] S. Yousefi , C. Mihalache , E. Kozlowski , I. Schmid , and H. U. Simon , “Viable Neutrophils Release Mitochondrial DNA to Form Neutrophil Extracellular Traps,” Cell Death and Differentiation 16, no. 11 (2009): 1438–1444. 19609275 10.1038/cdd.2009.96

[61] S. Yousefi , et al., “Catapult‐Like release of mitochondrial DNA by eosinophils contributes to antibacterial defense,” Nature Medicine 14, no. 9 (2008): 949–953. 10.1038/nm.1855 18690244

[62] D. Stojkov , P. Amini , and K. Oberson , “ROS and glutathionylation balance cytoskeletal dynamics in neutrophil extracellular trap formation,” Journal of Cell Biology 216, no. 12 (2017): 4073–4090. 29150539 10.1083/jcb.201611168 PMC5716265

[63] S. Yousefi , D. Stojkov , and N. Germic , “Untangling “NETosis” From NETs,” European Journal of Immunology 49, no. 2 (2019): 221–227. 30629284 10.1002/eji.201747053

[64] S. Peng , J. Gao , D. Stojkov , S. Yousefi , and H.‐U. Simon , “Established and emerging roles for mitochondria in neutrophils,” Immunological Reviews 314, no. 1 (2023): 413–426. 36331270 10.1111/imr.13158

[65] M. K. Thind , H. H. Uhlig , and M. Glogauer , “A metabolic perspective of the neutrophil life cycle: New avenues in immunometabolism,” Frontiers in immunology 14 (2023): 1334205. 38259490 10.3389/fimmu.2023.1334205 PMC10800387

[66] O. Rodríguez‐Espinosa , O. Rojas‐Espinosa , M. M. B. Moreno‐Altamirano , E. O. López‐Villegas , and F. J. Sánchez‐García , “Metabolic requirements for neutrophil extracellular traps formation,” Immunology 145, no. 2 (2015): 213–224. 25545227 10.1111/imm.12437 PMC4427386

[67] B. E. Hsu , S. Tabariès , and R. M. Johnson , “Immature Low‐Density Neutrophils Exhibit Metabolic Flexibility that Facilitates Breast Cancer Liver Metastasis,” Cell Reports 27, no. 13 (2019): 3902–3915.e6. 31242422 10.1016/j.celrep.2019.05.091

[68] E. C. Britt , J. Lika , and M. A. Giese , “Switching to the cyclic pentose phosphate pathway powers the oxidative burst in activated neutrophils,” Nature Metabolism 4, no. 3 (2022): 389–403. 10.1038/s42255-022-00550-8 PMC8964420 35347316

[69] D. Stojkov , L. Gigon , and S. Peng , “Physiological and Pathophysiological Roles of Metabolic Pathways for NET Formation and Other Neutrophil Functions,” Frontiers in immunology 13 (2022): 826515. 35251008 10.3389/fimmu.2022.826515 PMC8889909

[70] Y. Ye , Y. Wang , and Q. Xu , “In vitro study: HIF‐1α‐dependent glycolysis enhances NETosis in hypoxic conditions,” Frontiers in immunology 16 (2025): 1583587. 40356921 10.3389/fimmu.2025.1583587 PMC12066692

[71] D. N. Douda , L. Yip , M. A. Khan , H. Grasemann , and N. Palaniyar , “Akt is essential to induce NADPH‐dependent NETosis and to switch the neutrophil death to apoptosis,” Blood 123, no. 4 (2014): 597–600. 24458280 10.1182/blood-2013-09-526707

[72] M. A. Khan , A. Farahvash , and D. N. Douda , “JNK Activation Turns on LPS‐ and Gram‐Negative Bacteria‐Induced NADPH Oxidase‐Dependent Suicidal NETosis,” Scientific Reports 7, no. 1 (2017): 3409. 28611461 10.1038/s41598-017-03257-z PMC5469795

[73] A. Korba‐Mikołajczyk , K. D. Służalska , and P. Kasperkiewicz , “Exploring the involvement of serine proteases in neutrophil extracellular traps: A review of mechanisms and implications,” Cell death & disease 16, no. 1 (2025): 535. 40681487 10.1038/s41419-025-07857-w PMC12274431

[74] V. Papayannopoulos , K. D. Metzler , A. Hakkim , and A. Zychlinsky , “Neutrophil elastase and myeloperoxidase regulate the formation of neutrophil extracellular traps,” Journal of Cell Biology 191, no. 3 (2010): 677–691. 20974816 10.1083/jcb.201006052 PMC3003309

[75] K. D. Metzler , T. A. Fuchs , and W. M. Nauseef , “Myeloperoxidase is required for neutrophil extracellular trap formation: Implications for innate immunity,” Blood 117, no. 3 (2011): 953–959. 20974672 10.1182/blood-2010-06-290171 PMC3035083

[76] K. D. Metzler , C. Goosmann , A. Lubojemska , A. Zychlinsky , and V. Papayannopoulos , “A myeloperoxidase‐containing complex regulates neutrophil elastase release and actin dynamics During NETosis,” Cell reports 8, no. 3 (2014): 883–896. 25066128 10.1016/j.celrep.2014.06.044 PMC4471680

[77] P. Ettel and T. Weichhart , “Not just sugar: Metabolic control of neutrophil development and effector functions,” Journal of Leukocyte Biology 116, no. 3 (2024): 487–510.. 38450755 10.1093/jleuko/qiae057 PMC7617515

[78] Y. P. Zhu , M. Speir , and Z. Tan , “NET formation is a default epigenetic program controlled by PAD4 in apoptotic neutrophils,” Science Advances 9, no. 51 (2023): eadj1397. 38117877 10.1126/sciadv.adj1397 PMC10732518

[79] C. Thålin , K. Aguilera , and N. W. Hall , “Quantification of citrullinated histones: Development of an improved assay to reliably quantify nucleosomal H3Cit in human plasma,” Journal of Thrombosis and Haemostasis 18, no. 10 (2020): 2732–2743. 32654410 10.1111/jth.15003 PMC8722705

[80] Y. Matsuda , H. Hamayasu , and A. Seki , “Presence of Citrullinated Histone H3‐Positive Neutrophils in Microscopic Polyangiitis From the Early Phase: An Autopsy Proven Case,” Pathology International 66, no. 8 (2016): 466–471. 27427341 10.1111/pin.12434

[81] M. Leshner , S. Wang , and C. Lewis , “PAD4 mediated histone hypercitrullination induces heterochromatin decondensation and chromatin unfolding to form neutrophil extracellular trap‐Like structures,” Frontiers in immunology 3 (2012): 307. 23060885 10.3389/fimmu.2012.00307 PMC3463874

[82] H. D. Lewis , J. Liddle , and J. E. Coote , “Inhibition of PAD4 activity is sufficient to disrupt mouse and human NET formation,” Nature Chemical Biology 11, no. 3 (2015): 189–191. 25622091 10.1038/nchembio.1735 PMC4397581

[83] H. R. Thiam , S. L. Wong , and R. Qiu , “NETosis proceeds by cytoskeleton and endomembrane disassembly and PAD4‐mediated chromatin decondensation and nuclear envelope rupture,” PNAS 117, no. 13 (2020): 7326–7337. 32170015 10.1073/pnas.1909546117 PMC7132277

[84] J. Singh , L. Zlatar , and M. Muñoz‐Becerra , “Calpain‐1 weakens the nuclear envelope and promotes the release of neutrophil extracellular traps,” Cell Communication and Signaling 22, no. 1 (2024): 435. 39252008 10.1186/s12964-024-01785-6 PMC11384698

[85] S. Gößwein , et al., “Citrullination Licenses Calpain to Decondense Nuclei in Neutrophil Extracellular Trap Formation,” Frontiers in immunology 10 (2019): 2481. 31695698 10.3389/fimmu.2019.02481 PMC6817590

[86] E. F. Kenny , A. Herzig , and R. Krüger , “Diverse stimuli engage different neutrophil extracellular trap pathways,” Elife 6 (2017): e24437. 28574339 10.7554/eLife.24437 PMC5496738

[87] G. Franck , T. L. Mawson , and E. J. Folco , “Roles of PAD4 and NETosis in Experimental Atherosclerosis and Arterial Injury: Implications for Superficial Erosion,” Circulation Research 123, no. 1 (2018): 33–42. 29572206 10.1161/CIRCRESAHA.117.312494 PMC6014872

[88] L. Dejas , K. Santoni , E. Meunier , and M. Lamkanfi , “Regulated cell death in neutrophils: From apoptosis to NETosis and pyroptosis,” Seminars in Immunology 70 (2023): 101849. 37939552 10.1016/j.smim.2023.101849 PMC10753288

[89] D. Azzouz , M. A. Khan , and N. Palaniyar , “ROS induces NETosis by oxidizing DNA and initiating DNA repair,” Cell Death Discovery 7, no. 1 (2021): 113.. 34001856 10.1038/s41420-021-00491-3 PMC8128883

[90] T. Muralt , S. M. Buonomo , M. P. Tschan , and M. Schaller , “Autophagy, NET formation, and inflammation crosstalk in thrombotic autoimmune diseases,” Frontiers in immunology 17 (2026): 1844170. 42266695 10.3389/fimmu.2026.1844170 PMC13243073

[91] Y. Xie , J. Li , R. Kang , and D. Tang , “Interplay Between Lipid Metabolism and Autophagy,” Frontiers in Cell and Developmental Biology 8 (2020): 431. 32582708 10.3389/fcell.2020.00431 PMC7283384

[92] G. Sollberger , A. Choidas , and G. L. Burn , “Gasdermin D plays a vital role in the generation of neutrophil extracellular traps,” Science Immunology 3, no. 26 (2018): eaar6689. 30143555 10.1126/sciimmunol.aar6689

[93] K. W. Chen , M. Monteleone , and D. Boucher , “Noncanonical inflammasome signaling elicits gasdermin D‐dependent neutrophil extracellular traps,” Science Immunology 3, no. 26 (2018): eaar6676. 30143554 10.1126/sciimmunol.aar6676

[94] L. Zeng , W. Xiang , W. Xiao , Y. Wu , and L. Sun , “The emerging role of neutrophil extracellular traps in autoimmune and autoinflammatory diseases,” MedComm 6, no. 3 (2020): e70101. 10.1002/mco2.70101 PMC11885892 40060194

[95] D. Chauhan , D. Demon , and L. Vande Walle , “GSDMD drives canonical inflammasome‐induced neutrophil pyroptosis and is dispensable for NETosis,” Embo Reports 23, no. 10 (2022): e54277. 35899491 10.15252/embr.202154277 PMC9535806

[96] D. Stojkov , M. J. Claus , and E. Kozlowski , “NET formation is independent of gasdermin D and pyroptotic cell death,” Science signaling 16, no. 769 (2023): eabm0517. 36693132 10.1126/scisignal.abm0517

[97] Y. Wei , B. Lan , and T. Zheng , “GSDME‐mediated pyroptosis promotes the progression and associated inflammation of atherosclerosis,” Nature Communications 14, no. 1 (2023): 929. 10.1038/s41467-023-36614-w PMC9938904 36807553

[98] F. Ma , L. Ghimire , and Q. Ren , “Gasdermin E dictates inflammatory responses by controlling the mode of neutrophil death,” Nature Communications 15, no. 1 (2024): 386. 10.1038/s41467-023-44669-y PMC10776763 38195694

[99] M. Sano , Y. Maejima , and S. Nakagama , “Neutrophil extracellular traps‐mediated Beclin‐1 suppression aggravates atherosclerosis by inhibiting macrophage autophagy,” Frontiers in Cell and Developmental Biology 10 (2022): 876147. 35923856 10.3389/fcell.2022.876147 PMC9340257

[100] S. Yousefi , J. A. Gold , and N. Andina , “Endothelial cells and coagulation,” Cell and Tissue Research 387, no. 3 (2022): 391–398. 34014399 10.1007/s00441-021-03471-2 PMC8975780

[101] D. F. Noubouossie , B. N. Reeves , B. D. Strahl , and N. S. Key , “Neutrophils: Back in the thrombosis spotlight,” Blood 133, no. 20 (2019): 2186–2197. 30898858 10.1182/blood-2018-10-862243 PMC7218731

[102] D. A. Stakos , K. Kambas , and T. Konstantinidis , “Expression of functional tissue factor by neutrophil extracellular traps in culprit artery of acute myocardial infarction,” European Heart Journal 36, no. 22 (2015): 1405–1414. 25660055 10.1093/eurheartj/ehv007 PMC4458286

[103] E. L. G. Pryzdial , A. Leatherdale , and E. M. Conway , “Coagulation and complement: Key innate defense participants in a seamless web,” Frontiers in immunology 13 (2022): 918775.. 36016942 10.3389/fimmu.2022.918775 PMC9398469

[104] T.‐D. Tsourouktsoglou , A. Warnatsch , M. Ioannou , D. Hoving , Q. Wang , and V. Papayannopoulos , “Histones, DNA, and Citrullination Promote Neutrophil Extracellular Trap Inflammation by Regulating the Localization and Activation of TLR4,” Cell reports 31, no. 5 (2020): 107602. 32375035 10.1016/j.celrep.2020.107602

[105] Z. Liu , N. Huang , and C. Liu , “Mitochondrial DNA in atherosclerosis research progress: A mini review,” Frontiers in immunology 16 (2025): 1526390. 39991161 10.3389/fimmu.2025.1526390 PMC11842404

[106] H. Zhang , Y. Wang , and M. Qu , “Neutrophil, neutrophil extracellular traps and endothelial cell dysfunction in sepsis,” Clinical and translational medicine 13, no. 1 (2023): e1170. 36629024 10.1002/ctm2.1170 PMC9832433

[107] E. Yu , P. A. Calvert , and J. R. Mercer , “Mitochondrial DNA damage can promote atherosclerosis independently of reactive oxygen species Through effects on smooth muscle cells and monocytes and correlates With higher‐risk plaques in humans,” Circulation 128, no. 7 (2013): 702–712. 23841983 10.1161/CIRCULATIONAHA.113.002271

[108] N. R. Madamanchi and M. S. Runge , “Mitochondrial dysfunction in atherosclerosis,” Circulation Research 100, no. 4 (2007): 460–473.. 17332437 10.1161/01.RES.0000258450.44413.96

[109] D. J. Tyrrell , M. G. Blin , and J. Song , “Age‐Associated Mitochondrial Dysfunction Accelerates Atherogenesis,” Circulation Research 126, no. 3 (2020): 298–314. 31818196 10.1161/CIRCRESAHA.119.315644 PMC7006722

[110] M. V. Pinti , G. K. Fink , Q. A. Hathaway , A. J. Durr , A. Kunovac , and J. M. Hollander , “Mitochondrial dysfunction in type 2 diabetes mellitus: An organ‐based analysis,” American Journal of Physiology. Endocrinology and Metabolism 316, no. 2 (2019): E268–E285. 30601700 10.1152/ajpendo.00314.2018 PMC6397358

[111] O. J. de Boer , et al., “Neutrophils, neutrophil extracellular traps and interleukin‐17 associate With the organisation of thrombi in acute myocardial infarction,” Thrombosis and Haemostasis 109, no. 2 (2013): 290–297. 23238559 10.1160/TH12-06-0425

[112] M. Saffarzadeh , C. Juenemann , and M. A. Queisser , “Neutrophil extracellular traps directly induce epithelial and endothelial cell death: A predominant role of histones,” PLoS One 7, no. 2 (2012): e32366. 22389696 10.1371/journal.pone.0032366 PMC3289648

[113] Y. Wang , G. Z. Wang , P. S. Rabinovitch , and I. Tabas , “Macrophage mitochondrial oxidative stress promotes atherosclerosis and nuclear factor‐κB‐mediated inflammation in macrophages,” Circulation Research 114, no. 3 (2014): 421–433. 24297735 10.1161/CIRCRESAHA.114.302153 PMC3946745

[114] I. Varjú , A. Tanka‐Salamon , and K. Kolev , “Neutrophil Extracellular Traps: At the Interface of Thrombosis and Comorbidities,” Seminars in Thrombosis and Hemostasis 51, no. 7 (2025): 724–735.. 40020757 10.1055/a-2548-0805

[115] Z. An , J. Li , and J. Yu , “Neutrophil extracellular traps induced by IL‐8 aggravate atherosclerosis via activation NF‐κB signaling in macrophages,” Cell Cycle 18, no. 21 (2019): 2928–2938. 31496351 10.1080/15384101.2019.1662678 PMC6791689

[116] R. Arroyo Hornero , R. A. Maqueda‐Alfaro , and M. A. Solís‐Barbosa , “TNF and type I interferon crosstalk controls the fate and function of plasmacytoid dendritic cells,” Nature Immunology 26, no. 9 (2025): 1540–1552. 40796660 10.1038/s41590-025-02234-3 PMC12396960

[117] Y. Döring , et al., “Auto‐antigenic protein‐DNA complexes stimulate plasmacytoid dendritic cells to promote atherosclerosis,” Circulation 125, no. 13 (2012): 1673–1683. 22388324 10.1161/CIRCULATIONAHA.111.046755

[118] C. N. Morrell , Z. T. Hilt , D. N. Pariser , and P. Maurya , “PAD4 and von Willebrand Factor Link Inflammation and Thrombosis,” Circulation Research 125, no. 5 (2019): 520–522. 31415230 10.1161/CIRCRESAHA.119.315601 PMC10198561

[119] N. Sorvillo , D. M. Mizurini , and C. Coxon , “Plasma Peptidylarginine Deiminase IV Promotes VWF‐Platelet String Formation and Accelerates Thrombosis After Vessel Injury,” Circulation Research 125, no. 5 (2019): 507–519. 31248335 10.1161/CIRCRESAHA.118.314571 PMC6697196

[120] W. Zhang , Y. Zhang , and L. Han , “Double‐stranded DNA enhances platelet activation, thrombosis, and myocardial injury via cyclic GMP‐AMP synthase,” Cardiovascular Research 121, no. 2 (2025): 353–366. 39302147 10.1093/cvr/cvae218

[121] J. Cao , S. Roth , and S. Zhang , “DNA‐sensing inflammasomes cause recurrent atherosclerotic stroke,” Nature 633, no. 8029 (2024): 433–441. 39112714 10.1038/s41586-024-07803-4 PMC11390481

[122] S. Sinha , W. Watorek , S. Karr , J. Giles , W. Bode , and J. Travis , “Primary structure of human neutrophil elastase,” PNAS 84, no. 8 (1987): 2228–2232. 3550808 10.1073/pnas.84.8.2228 PMC304622

[123] K. Kambas , A. Chrysanthopoulou , and D. Vassilopoulos , “Tissue factor expression in neutrophil extracellular traps and neutrophil derived microparticles in antineutrophil cytoplasmic antibody associated vasculitis may promote thromboinflammation and the thrombophilic state associated With the disease,” Annals of the Rheumatic Diseases 73, no. 10 (2014): 1854–1863. 23873874 10.1136/annrheumdis-2013-203430

[124] S. Massberg , L. Grahl , and M.‐L. von Bruehl , “Reciprocal coupling of coagulation and innate immunity via neutrophil serine proteases,” Nature Medicine 16, no. 8 (2010): 887–896. 10.1038/nm.2184 20676107

[125] P. L. Jackson , X. Xu , and L. Wilson , “Human neutrophil elastase‐mediated cleavage sites of MMP‐9 and TIMP‐1: Implications to cystic fibrosis proteolytic dysfunction,” Molecular Medicine 16, no. 5‐6 (2010): 159–166. 20111696 10.2119/molmed.2009.00109 PMC2811559

[126] H. Manoj , S. M. Gomes , P. Y. Thimmappa , P. R. Nagareddy , C. Jamora , and M. B. Joshi , “Cytokine signalling in formation of neutrophil extracellular traps: Implications for health and diseases,” Cytokine & Growth Factor Reviews 81 (2025): 27–39. 39681501 10.1016/j.cytogfr.2024.12.001

[127] G. R. Sambrano , W. Huang , T. Faruqi , S. Mahrus , C. Craik , and S. R. Coughlin , “Cathepsin G activates protease‐activated receptor‐4 in human platelets,” Journal of Biological Chemistry 275, no. 10 (2000): 6819–6823. 10702240 10.1074/jbc.275.10.6819

[128] E. J. Folco , T. L. Mawson , and A. Vromman , “Neutrophil Extracellular Traps Induce Endothelial Cell Activation and Tissue Factor Production Through Interleukin‐1α and Cathepsin G,” Arteriosclerosis, Thrombosis, and Vascular Biology 38, no. 8 (2018): 1901–1912. 29976772 10.1161/ATVBAHA.118.311150 PMC6202190

[129] T. Quillard , H. A. Araújo , G. Franck , E. Shvartz , G. Sukhova , and P. Libby , “TLR2 and neutrophils potentiate endothelial stress, apoptosis and detachment: Implications for superficial erosion,” European Heart Journal 36, no. 22 (2015): 1394–1404. 25755115 10.1093/eurheartj/ehv044 PMC4458287

[130] R. Boxio , J. Wartelle , and B. Nawrocki‐Raby , “Neutrophil elastase cleaves epithelial cadherin in acutely injured lung epithelium,” Respiratory Research 17, no. 1 (2016): 129. 27751187 10.1186/s12931-016-0449-x PMC5067913

[131] Y. Aratani , “Myeloperoxidase: Its role for host defense, inflammation, and neutrophil function,” Archives of Biochemistry and Biophysics 640 (2018): 47–52.. 29336940 10.1016/j.abb.2018.01.004

[132] H. J. Hamam and N. Palaniyar , “Post‐Translational Modifications in NETosis and NETs‐Mediated Diseases,” Biomolecules 9, no. 8 (2019): 369. 31416265 10.3390/biom9080369 PMC6723044

[133] S. J. Nicholls and S. L. Hazen , “Myeloperoxidase and cardiovascular disease,” Arteriosclerosis, Thrombosis, and Vascular Biology 25, no. 6 (2005): 1102–1111. 15790935 10.1161/01.ATV.0000163262.83456.6d

[134] B. Shao , M. N. Oda , and C. Bergt , “Myeloperoxidase impairs ABCA1‐dependent cholesterol efflux Through methionine oxidation and site‐specific tyrosine chlorination of apolipoprotein A‐I,” Journal of Biological Chemistry 281, no. 14 (2006): 9001–9004. 16497665 10.1074/jbc.C600011200

[135] N. Teng , G. J. Maghzal , J. Talib , I. Rashid , A. K. Lau , and R. Stocker , “The roles of myeloperoxidase in coronary artery disease and its potential implication in plaque rupture,” Redox Report: Communications in Free Radical Research 22, no. 2 (2017): 51–73. 27884085 10.1080/13510002.2016.1256119 PMC6837458

[136] Y. Chen , X. Li , and X. Lin , “Complement C5a induces the generation of neutrophil extracellular traps by inhibiting mitochondrial STAT3 to promote the development of arterial thrombosis,” Thrombosis Journal 20, no. 1 (2022): 24. 35488279 10.1186/s12959-022-00384-0 PMC9051782

[137] A. Wezel , M. R. de Vries , and H. M. Lagraauw , “Complement factor C5a induces atherosclerotic plaque disruptions,” Journal of Cellular and Molecular Medicine 18, no. 10 (2014): 2020–2030. 25124749 10.1111/jcmm.12357 PMC4244017

[138] A. C. Newby , “Metalloproteinase production From macrophages—a perfect storm leading to atherosclerotic plaque rupture and myocardial infarction,” Experimental Physiology 101, no. 11 (2016): 1327–1337. 26969796 10.1113/EP085567

[139] R. Zhang , C. Sun , and Y. Han , “Neutrophil autophagy and NETosis in COVID‐19: Perspectives,” Autophagy 19, no. 3 (2023): 758–767. 35951555 10.1080/15548627.2022.2099206 PMC9980466

[140] X. Zhang , H. Song , and D. Liu , “S100A12 triggers NETosis to aggravate myocardial infarction injury via the Annexin A5‐calcium axis,” Nature Communications 16, no. 1 (2025): 1746. 10.1038/s41467-025-56978-5 PMC11836404 39966386

[141] S. Hu , F. Zhang , and J. Wang , “MMP9(High) Neutrophils are Critical Mediators of Neutrophil Extracellular Traps Formation and Myocardial Ischemia/Reperfusion Injury,” Advanced science (Weinheim, Baden‐Wurttemberg, Germany) 12, no. 21 (2025): e2415205. 40151877 10.1002/advs.202415205 PMC12140383

[142] S. Lenglet , F. Mach , and F. Montecucco , “Role of matrix metalloproteinase‐8 in atherosclerosis,” Mediators of Inflammation 2013 (2013): 1–6.. 10.1155/2013/659282 PMC3556866 23365489

[143] T. Josefs , T. J. Barrett , and E. J. Brown , “Neutrophil extracellular traps promote macrophage inflammation and impair atherosclerosis resolution in diabetic mice,” JCI Insight 5, no. 7 (2020): e134796. 32191637 10.1172/jci.insight.134796 PMC7205252

[144] T. Karasawa and M. Takahashi , “Inflammasome Activation and Neutrophil Extracellular Traps in Atherosclerosis,” Journal of Atherosclerosis and Thrombosis 32, no. 5 (2025): 535–549.. 39828369 10.5551/jat.RV22033 PMC12055512

[145] J. Li , Z. Wang , J. Li , H. Zhao , and Q. Ma , “HMGB1: A New Target for Ischemic Stroke and Hemorrhagic Transformation,” Translational Stroke Research 16, no. 3 (2025): 990–1015. 38740617 10.1007/s12975-024-01258-5 PMC12045843

[146] X. Wang , M. Mayorga‐Flores , K. G. Bien , A. O. Bailey , and J. Iwahara , “DNA‐mediated proteolysis by neutrophil elastase enhances binding activities of the HMGB1 protein,” Journal of Biological Chemistry 298, no. 11 (2022): 102577. 36220391 10.1016/j.jbc.2022.102577 PMC9664404

[147] N. Maugeri , L. Campana , and M. Gavina , “Activated platelets present high mobility group box 1 to neutrophils, inducing autophagy and promoting the extrusion of neutrophil extracellular traps,” Journal of Thrombosis and Haemostasis 12, no. 12 (2014): 2074–2088. 25163512 10.1111/jth.12710

[148] M. Kinoshita , Y. Ogawa , and N. Hama , “Neutrophils initiate and exacerbate Stevens‐Johnson syndrome and toxic epidermal necrolysis,” Science Translational Medicine 13, no. 600 (2021): eaax2398. 34193610 10.1126/scitranslmed.aax2398 PMC9392155

[149] S.‐W. Kim , H. Lee , H.‐K. Lee , I.‐D. Kim , and J.‐K. Lee , “Neutrophil extracellular trap induced by HMGB1 exacerbates damages in the ischemic brain,” Acta Neuropathologica Communications 7, no. 1 (2019): 94. 31177989 10.1186/s40478-019-0747-x PMC6556959

[150] A. Bangert , M. Andrassy , and A.‐M. Müller , “Critical role of RAGE and HMGB1 in inflammatory heart disease,” PNAS 113, no. 2 (2016): E155–E164. 26715748 10.1073/pnas.1522288113 PMC4720305

[151] A. Neumann , E. T. M. Berends , and A. Nerlich , “The antimicrobial peptide LL‐37 facilitates the formation of neutrophil extracellular traps,” Biochemical Journal 464, no. 1 (2014): 3–11. 25181554 10.1042/BJ20140778

[152] R. Lande , D. Ganguly , and V. Facchinetti , “Neutrophils activate plasmacytoid dendritic cells by releasing self‐DNA‐peptide complexes in systemic lupus erythematosus,” Science Translational Medicine 3, no. 73 (2011): 73ra19. 10.1126/scitranslmed.3001180 PMC3399524 21389263

[153] N. Mookherjee , M. A. Anderson , H. P. Haagsman , and D. J. Davidson , “Antimicrobial host defence peptides: Functions and clinical potential,” Nature Reviews Drug Discovery 19, no. 5 (2020): 311–332. 32107480 10.1038/s41573-019-0058-8

[154] C. D. Ciornei , H. Tapper , A. Bjartell , N. H. Sternby , and M. Bodelsson , “Human antimicrobial peptide LL‐37 is present in atherosclerotic plaques and induces death of vascular smooth muscle cells: A laboratory study,” BMC Cardiovascular Disorders [Electronic Resource] 6 (2006): 49. 17181861 10.1186/1471-2261-6-49 PMC1764755

[155] Z. Duan , J. Zhang , and X. Chen , “Role of LL‐37 in thrombotic complications in patients With COVID‐19,” Cellular and Molecular Life Sciences 79, no. 6 (2022): 309. 35596804 10.1007/s00018-022-04309-y PMC9123294

[156] P. Kougias , H. Chai , P. H. Lin , Q. Yao , A. B. Lumsden , and C. Chen , “Neutrophil antimicrobial peptide alpha‐defensin causes endothelial dysfunction in porcine coronary arteries,” Journal of Vascular Surgery 43, no. 2 (2006): 357–363. 16476615 10.1016/j.jvs.2005.10.019

[157] R. Abu‐Fanne , E. Maraga , and I. Abd‐Elrahman , “α‐Defensins Induce a Post‐translational Modification of Low Density Lipoprotein (LDL) That Promotes Atherosclerosis at Normal Levels of Plasma Cholesterol,” Journal of Biological Chemistry 291, no. 6 (2016): 2777–2786. 26518877 10.1074/jbc.M115.669812 PMC4742743

[158] M. Shapira , A. Roguin , and I. Fayad , “Predictive value of baseline alpha defensin level in patients With stable coronary artery disease: A retrospective single center study,” IJC Heart & Vasculature 53 (2024): 101465. 39091435 10.1016/j.ijcha.2024.101465 PMC11292519

[159] J. W. Xie , Y. Wang , and K. Xiao , “Alpha defensin‐1 attenuates surgically induced osteoarthritis in association With promoting M1 to M2 macrophage polarization,” Osteoarthritis and Cartilage 29, no. 7 (2021): 1048–1059. 33892137 10.1016/j.joca.2021.04.006

[160] F. Katkat , S. Varol , and N. Isiksacan , “Human neutrophil peptides 1–3 level in patients With acute myocardial infarction and its relation With coronary artery disease severity,” Turk Kardiyoloji Dernegi Arsivi 49, no. 2 (2021): 120–126. 33709917 10.5543/tkda.2021.99537

[161] A. R. Tall and M. Westerterp , “Inflammasomes, neutrophil extracellular traps, and cholesterol,” Journal of Lipid Research 60, no. 4 (2019): 721–727.. 30782961 10.1194/jlr.S091280 PMC6446695

[162] X. Shan , X. Fan , X. Geng , Y. Liang , Y. Yang , and J. Zhang , “Interactions Between neutrophil extracellular traps and macrophages: The key to inflammatory diseases,” Frontiers in immunology 17 (2026): 1731687. 41869325 10.3389/fimmu.2026.1731687 PMC13002455

[163] C. Silvestre‐Roig , Q. Braster , and K. Wichapong , “Externalized histone H4 orchestrates chronic inflammation by inducing lytic cell death,” Nature 569, no. 7755 (2019): 236–240. 31043745 10.1038/s41586-019-1167-6 PMC6716525

[164] R. T. A. Megens , S. Vijayan , and D. Lievens , “Presence of luminal neutrophil extracellular traps in atherosclerosis,” Thrombosis and Haemostasis 107, no. 3 (2012): 597–598. 22318427 10.1160/TH11-09-0650

[165] J. Thazhathveettil , A. K. Kumawat , I. Demirel , A. Sirsjö , and G. V. Paramel , “Vascular smooth muscle cells in response to cholesterol crystals modulates inflammatory cytokines release and promotes neutrophil extracellular trap formation,” Molecular Medicine 30, no. 1 (2024): 42. 38519881 10.1186/s10020-024-00809-8 PMC10960408

[166] Y. G. Zhang , Y. Song , and X.‐L. Guo , “Exosomes derived From oxLDL‐stimulated macrophages induce neutrophil extracellular traps to drive atherosclerosis,” Cell Cycle 18, no. 20 (2019): 2672–2682. 10.1080/15384101.2019.1654797 PMC6773244 31416388

[167] W. Liu , B. D. Hardaway , and E. Kim , “Inflammatory crosstalk impairs phagocytic receptors and aggravates atherosclerosis in clonal hematopoiesis in mice,” Journal of Clinical Investigation 135, no. 1 (2025): e182939. 10.1172/JCI182939 PMC11684819 39531316

[168] Y. Cao , M. Chen , and X. Jiao , “Neutrophil extracellular traps mediate the crosstalk Between plaque microenvironment and unstable carotid plaque formation,” Experimental & Molecular Medicine 56, no. 8 (2024): 1717–1735. 39085350 10.1038/s12276-024-01281-4 PMC11372095

[169] Y. Zhu , T. Wang , and Y. Yang , “Low shear stress exacerbates atherosclerosis by inducing the generation of neutrophil extracellular traps via Piezo1‐mediated mechanosensation,” Atherosclerosis 391 (2024): 117473. 38412763 10.1016/j.atherosclerosis.2024.117473

[170] J. S. Knight , W. Luo , and A. A. O'Dell , “Peptidylarginine deiminase inhibition reduces vascular damage and modulates innate immune responses in murine models of atherosclerosis,” Circulation Research 114, no. 6 (2014): 947–956. 24425713 10.1161/CIRCRESAHA.114.303312 PMC4185401

[171] J. S. Knight , W. Zhao , and W. Luo , “Peptidylarginine deiminase inhibition is immunomodulatory and vasculoprotective in murine lupus,” Journal of Clinical Investigation 123, no. 7 (2013): 2981–2993. 23722903 10.1172/JCI67390 PMC3696545

[172] R. Vergallo and F. Crea , “Atherosclerotic Plaque Healing,” New England Journal of Medicine 383, no. 9 (2020): 846–857.. 32846063 10.1056/NEJMra2000317

[173] J. F. Bentzon , F. Otsuka , R. Virmani , and E. Falk , “Mechanisms of plaque formation and rupture,” Circulation Research 114, no. 12 (2014): 1852–1866. 24902970 10.1161/CIRCRESAHA.114.302721

[174] A. Mangold , S. Alias , and T. Scherz , “Coronary neutrophil extracellular trap burden and deoxyribonuclease activity in ST‐elevation acute coronary syndrome are predictors of ST‐segment resolution and infarct size,” Circulation Research 116, no. 7 (2015): 1182–1192. 25547404 10.1161/CIRCRESAHA.116.304944

[175] T. Adriaenssens and R. Byrne , “PREvention of late Stent Thrombosis by an Interdisciplinary Global European effort: PRESTIGE,” European Heart Journal 35, no. 32 (2014): 2128–2129.. 25289392

[176] J. Riegger , R. A. Byrne , and M. Joner , “Histopathological evaluation of thrombus in patients presenting With stent thrombosis. A multicenter European study: A report of the prevention of late stent thrombosis by an interdisciplinary global European effort consortium,” European Heart Journal 37, no. 19 (2016): 1538–1549. 26761950 10.1093/eurheartj/ehv419 PMC4872283

[177] S. Kapoor , L. Mihalovičová , and E. Pisareva , “Association of vascular netosis With COVID‐19 severity in asymptomatic and symptomatic patients,” Iscience 27, no. 5 (2024): 109573. 38660409 10.1016/j.isci.2024.109573 PMC11039348

[178] Z. Han , J. Xu , and M. Yue , “Molecular Mechanisms of NETs and Platelet Interactions and Their Regulatory Functions in Sepsis,” Scandinavian Journal of Immunology 103, no. 5 (2026): e70120.. 42141379 10.1111/sji.70120

[179] D. Kolte , T. Yonetsu , J. C. Ye , P. Libby , V. Fuster , and I.‐K. Jang , “Optical Coherence Tomography of Plaque Erosion: JACC Focus Seminar Part 2/3,” Journal of the American College of Cardiology 78, no. 12 (2021): 1266–1274. 34531028 10.1016/j.jacc.2021.07.030

[180] K. R. Pertiwi , et al., “Neutrophil Extracellular Traps Participate in All Different Types of Thrombotic and Haemorrhagic Complications of Coronary Atherosclerosis,” Thrombosis and Haemostasis 118, no. 6 (2018): 1078–1087. 29672788 10.1055/s-0038-1641749

[181] J. Dai , L. Xing , and H. Jia , “In Vivo Predictors of Plaque Erosion in Patients With ST‐segment Elevation Myocardial Infarction: A Clinical, Angiographical, and Intravascular Optical Coherence Tomography Study,” European Heart Journal 39, no. 22 (2018): 2077–2085. 29547992 10.1093/eurheartj/ehy101

[182] L. Ge , X. Zhou , and W. Ji , “Neutrophil Extracellular Traps in Ischemia‐reperfusion Injury‐induced Myocardial no‐reflow: Therapeutic Potential of DNase‐based Reperfusion Strategy,” American Journal of Physiology. Heart and Circulatory Physiology 308, no. 5 (2015): H500–H509. 25527775 10.1152/ajpheart.00381.2014

[183] A. S. Savchenko , J. I. Borissoff , and K. Martinod , “VWF‐mediated Leukocyte Recruitment With Chromatin Decondensation by PAD4 Increases Myocardial Ischemia/Reperfusion Injury in Mice,” Blood 123, no. 1 (2014): 141–148. 24200682 10.1182/blood-2013-07-514992 PMC3879903

[184] G. Wigerblad and M. Kaplan , “Neutrophil Extracellular Traps in Systemic Autoimmune and Autoinflammatory Diseases,” Nature Reviews Immunology 23, no. 5 (2023): 274–288. 10.1038/s41577-022-00787-0 PMC9579530 36257987

[185] T. M. Hofbauer , A. Mangold , and T. Scherz , “Neutrophil Extracellular Traps and Fibrocytes in ST‐segment Elevation Myocardial Infarction,” Basic Research in Cardiology 114, no. 5 (2019): 33. 31312919 10.1007/s00395-019-0740-3 PMC6647191

[186] A. Chrysanthopoulou , I. Mitroulis , and E. Apostolidou , “Neutrophil Extracellular Traps Promote Differentiation and Function of Fibroblasts,” Journal of Pathology 233, no. 3 (2014): 294–307. 24740698 10.1002/path.4359

[187] L. Ciortan , R. D. Macarie , and E. Barbu , “Cross‐Talk between Neutrophils and Macrophages Post‐Myocardial Infarction: From Inflammatory Drivers to Therapeutic Targets,” International Journal of Molecular Sciences 26, no. 21 (2025): 10575. 41226611 10.3390/ijms262110575 PMC12607372

[188] K. Eghbalzadeh , L. Georgi , and T. Louis , “Compromised Anti‐inflammatory Action of Neutrophil Extracellular Traps in PAD4‐Deficient Mice Contributes to Aggravated Acute Inflammation after Myocardial Infarction,” Frontiers in immunology 10 (2019): 2313. 31632398 10.3389/fimmu.2019.02313 PMC6779806

[189] Y. Shao , Z. Guo , and Y. Yang , “Neutrophil Extracellular Traps Contribute to Myofibroblast Differentiation and Scar Hyperplasia Through the Toll‐Like Receptor 9/Nuclear Factor Kappa‐B/Interleukin‐6 Pathway,” Burns Trauma 10 (2022): tkac044. 36406661 10.1093/burnst/tkac044 PMC9668674

[190] M. S. Langseth , G. Ø. Andersen , and T. Husebye , “Neutrophil Extracellular Trap Components and Myocardial Recovery in Post‐ischemic Acute Heart Failure,” PLoS One 15, no. 10 (2020): e0241333. 33119664 10.1371/journal.pone.0241333 PMC7595325

[191] R. Helseth , S. Solheim , H. Arnesen , I. Seljeflot , and T. B. Opstad , “The Time Course of Markers of Neutrophil Extracellular Traps in Patients Undergoing Revascularisation for Acute Myocardial Infarction or Stable Angina Pectoris,” Mediators of Inflammation 2016 (2016): 2182358. 28074081 10.1155/2016/2182358 PMC5198181

[192] A. Mangold , A. S. Ondracek , and T. M. Hofbauer , “Culprit Site Extracellular DNA and Microvascular Obstruction in ST‐elevation Myocardial Infarction,” Cardiovascular Research 118, no. 8 (2022): 2006–2017. 34173822 10.1093/cvr/cvab217

[193] M. S. Langseth , R. Helseth , and V. Ritschel , “Double‐Stranded DNA and NETs Components in Relation to Clinical Outcome after ST‐Elevation Myocardial Infarction,” Scientific Reports 10, no. 1 (2020): 5007. 32193509 10.1038/s41598-020-61971-7 PMC7081350

[194] A. Blasco , A. Rosell , and R. Castejón , “Inflammatory and Neutrophil Extracellular Trap Markers to Predict Cardiac Events After ST‐segment Elevation Myocardial Infarction,” PLoS One 20, no. 4 (2025): e0319759. 40168324 10.1371/journal.pone.0319759 PMC11960995

[195] A. Blasco , A. Rosell , and R. Castejón , “Analysis of NETs (neutrophil extracellular traps) in Coronary Thrombus and Peripheral Blood of Patients With ST‐segment Elevation Myocardial Infarction,” Thrombosis Research 235 (2024): 18–21. 38281441 10.1016/j.thromres.2024.01.015

[196] S. Yang , Y. Xiao , and Y. Du , “Diagnostic and Prognostic Value of Neutrophil Extracellular Trap Levels in Patients with Acute Aortic Dissection,” Frontiers in Cardiovascular Medicine 8 (2021): 683445. 35242817 10.3389/fcvm.2021.683445 PMC8885526

[197] J. Natorska , M. Ząbczyk , and A. Undas , “Neutrophil Extracellular Traps (NETs) in Cardiovascular Diseases: From Molecular Mechanisms to Therapeutic Interventions,” Kardiologia Polska 81, no. 12 (2023): 1205–1216. 38189504 10.33963/v.kp.98520

[198] P. Mołek , et al., “Markers of NET Formation and Stroke Risk in Patients With Atrial Fibrillation: Association With a Prothrombotic state,” Thrombosis Research 213 (2022): 1–7. 35276507 10.1016/j.thromres.2022.02.025

[199] J. Torres‐Ruiz , A. Absalón‐Aguilar , and M. Nuñez‐Aguirre , “Neutrophil Extracellular Traps Contribute to COVID‐19 Hyperinflammation and Humoral Autoimmunity,” Cells 10, no. 10 (2021): 2545. 34685525 10.3390/cells10102545 PMC8533917

[200] T. M. Hofbauer , A. S. Ondracek , and A. Mangold , “Neutrophil Extracellular Traps Induce MCP‐1 at the Culprit Site in ST‐Segment Elevation Myocardial Infarction,” Frontiers in Cell and Developmental Biology 8 (2020): 564169. 33240874 10.3389/fcell.2020.564169 PMC7680894

[201] L. He , R. Liu , and H. Yue , “NETs Promote Pathogenic Cardiac Fibrosis and Participate in Ventricular Aneurysm Formation After Ischemia Injury Through the Facilitation of Perivascular Fibrosis,” Biochemical and Biophysical Research Communications 583 (2021): 154–161. 34735877 10.1016/j.bbrc.2021.10.068

[202] K. Traeuble , M. Munz , and J. Pauli , “Integrated Single‐cell Atlas of human Atherosclerotic Plaques,” Nature Communications 16, no. 1 (2025): 8255. 10.1038/s41467-025-63202-x PMC12423310 40931012

[203] J. Pauli , D. Garger , and F. Peymani , “Single Cell Spatial Transcriptomics Integration Deciphers the Morphological Heterogeneity of Atherosclerotic Carotid Arteries,” Nature Communications 16, no. 1 (2025): 11282. 10.1038/s41467-025-67679-4 PMC12717225 41413386

[204] M. S. Langseth , T. B. Opstad , and V. Bratseth , “Markers of Neutrophil Extracellular Traps Are Associated With Adverse Clinical Outcome in Stable Coronary Artery Disease,” European Journal of Preventive Cardiology 25, no. 7 (2018): 762–769. 29473463 10.1177/2047487318760618

[205] J. Y. T. Yiu , K. E. Hally , P. D. Larsen , and A. S. Holley , “Neutrophil‐Enriched Biomarkers and Long‐Term Prognosis in Acute Coronary Syndrome: A Systematic Review and Meta‐analysis,” Journal of Cardiovascular Translational Research 17, no. 2 (2024): 426–447. 37594719 10.1007/s12265-023-10425-2 PMC11052791

[206] X. Wang , D. Yang , J. Liu , X. Fan , A. Ma , and P. Liu , “Prognostic Value of Culprit Artery Double‐stranded DNA in ST‐segment Elevated Myocardial Infarction,” Scientific Reports 8, no. 1 (2018): 9294. 29915211 10.1038/s41598-018-27639-z PMC6006372

[207] T. M. Hofbauer , A. Mangold , and A. S. Ondracek , “Deoxyribonuclease 1 Q222R Single Nucleotide Polymorphism and Long‐term Mortality After Acute Myocardial Infarction,” Basic Research in Cardiology 116, no. 1 (2021): 29. 33891165 10.1007/s00395-021-00864-w PMC8064981

[208] K. Zabieglo , P. Majewski , and M. Majchrzak‐Gorecka , “The Inhibitory Effect of Secretory Leukocyte Protease Inhibitor (SLPI) on Formation of Neutrophil Extracellular Traps,” Journal of Leukocyte Biology 98, no. 1 (2015): 99–106. 25917460 10.1189/jlb.4AB1114-543R PMC4467168

[209] G. V. Haute , E. Caberlon , and E. Squizani , “Gallic Acid Reduces the Effect of LPS on Apoptosis and Inhibits the Formation of Neutrophil Extracellular Traps,” Toxicology in Vitro 30, no. 1 Pt B (2015): 309–317. 26475966 10.1016/j.tiv.2015.10.005

[210] X. Yu and S. Diamond , “Fibrin Modulates Shear‐Induced NETosis in Sterile Occlusive Thrombi Formed Under Haemodynamic Flow,” Thrombosis and Haemostasis 119, no. 4 (2019): 586–593. 30722079 10.1055/s-0039-1678529 PMC7046313

[211] F. Denorme , I. Portier , and J. L. Rustad , “Neutrophil Extracellular Traps Regulate Ischemic Stroke Brain Injury,” Journal of Clinical Investigation 132, no. 10 (2022): e154225. 35358095 10.1172/JCI154225 PMC9106355

[212] C. C. Yost , H. Schwertz , and M. J. Cody , “Neonatal NET‐inhibitory Factor and Related Peptides Inhibit Neutrophil Extracellular Trap Formation,” Journal of Clinical Investigation 126, no. 10 (2016): 3783–3798. 27599294 10.1172/JCI83873 PMC5096809

[213] H. Zhu , Z. Jian , and Y. Zhong , “Janus Kinase Inhibition Ameliorates Ischemic Stroke Injury and Neuroinflammation through Reducing NLRP3 Inflammasome Activation via JAK2/STAT3 Pathway Inhibition,” Frontiers in immunology 12 (2021): 714943. 34367186 10.3389/fimmu.2021.714943 PMC8339584

[214] W. M. Al‐Ghoul , M. S. Kim , N. Fazal , A. C. Azim , and A. Ali , “Evidence for Simvastatin Anti‐inflammatory Actions Based on Quantitative Analyses of NETosis and Other Inflammation/Oxidation Markers,” Results in Immunology 4 (2014): 14–22. 24809006 10.1016/j.rinim.2014.03.001 PMC4009405

[215] M. Cimino , W. Balduini , and S. Carloni , “Neuroprotective Effect of Simvastatin in Stroke: A Comparison Between Adult and Neonatal Rat Models of Cerebral Ischemia,” Neurotoxicology 26, no. 5 (2005): 929–933. 15923039 10.1016/j.neuro.2005.03.009

[216] E. Sapey , J. M. Patel , and H. Greenwood , “Simvastatin Improves Neutrophil Function and Clinical Outcomes in Pneumonia. A Pilot Randomized Controlled Clinical Trial,” American Journal of Respiratory and Critical Care Medicine 200, no. 10 (2019): 1282–1293. 31206313 10.1164/rccm.201812-2328OC PMC6857486

[217] R. Molinaro , et al., “Targeted Delivery of Protein Arginine Deiminase‐4 Inhibitors to Limit Arterial Intimal NETosis and Preserve Endothelial Integrity,” Cardiovascular Research 117, no. 13 (2021): 2652–2663. 33751034 10.1093/cvr/cvab074 PMC8783386

[218] C. Peña‐Martínez , et al., “Pharmacological Modulation of Neutrophil Extracellular Traps Reverses Thrombotic Stroke tPA (Tissue‐Type Plasminogen Activator) Resistance,” Stroke 50, no. 11 (2019): 3228–3237. 31526124 10.1161/STROKEAHA.119.026848

[219] V. Tiyerili , B. Camara , and M. U. Becher , “Neutrophil‐derived Myeloperoxidase Promotes Atherogenesis and Neointima Formation in Mice,” International Journal of Cardiology 204 (2016): 29–36. 26655530 10.1016/j.ijcard.2015.11.128

[220] W. Chen , S. Tumanov , and S. M. Kong , “Therapeutic Inhibition of MPO Stabilizes Pre‐existing High Risk Atherosclerotic Plaque,” Redox Biology 58 (2022): 102532. 36375379 10.1016/j.redox.2022.102532 PMC9663534

[221] T. Inghardt , T. Antonsson , and C. Ericsson , “Discovery of AZD4831, a Mechanism‐Based Irreversible Inhibitor of Myeloperoxidase, as a Potential Treatment for Heart Failure With Preserved Ejection Fraction,” Journal of Medicinal Chemistry 65, no. 17 (2022): 11485–11496. 36005476 10.1021/acs.jmedchem.1c02141 PMC9469207

[222] L. Gan , M. Lagerström‐Fermér , and H. Ericsson , “Safety, Tolerability, Pharmacokinetics and Effect on Serum Uric Acid of the Myeloperoxidase Inhibitor AZD4831 in a Randomized, Placebo‐controlled, Phase I Study in Healthy Volunteers,” British Journal of Clinical Pharmacology 85, no. 4 (2019): 762–770. 30618054 10.1111/bcp.13855 PMC6422671

[223] J. Huang , D. B. Agus , and C. J. Winfree , “Dehydroascorbic Acid, a Blood‐brain Barrier Transportable Form of Vitamin C, Mediates Potent Cerebroprotection in Experimental Stroke,” PNAS 98, no. 20 (2001): 11720–11724. 11573006 10.1073/pnas.171325998 PMC58796

[224] B. Mohammed , B. Fisher , and D. Kraskauskas , “Vitamin C: A Novel Regulator of Neutrophil Extracellular Trap Formation,” Nutrients 5, no. 8 (2013): 3131–3151. 23939536 10.3390/nu5083131 PMC3775246

[225] S. Nagel , J. Genius , S. Heiland , S. Horstmann , H. Gardner , and S. Wagner , “Diphenyleneiodonium and Dimethylsulfoxide for Treatment of Reperfusion Injury in Cerebral Ischemia of the Rat,” Brain Research 1132, no. 1 (2007): 210–217. 17184751 10.1016/j.brainres.2006.11.023

[226] Y. Huang , X. Zhang , and C. Zhang , “Edaravone Dexborneol Downregulates Neutrophil Extracellular Trap Expression and Ameliorates Blood‐Brain Barrier Permeability in Acute Ischemic Stroke,” Mediators of Inflammation 2022 (2022): 3855698. 36032782 10.1155/2022/3855698 PMC9410976

[227] A. O. Samokhin , P. A. Lythgo , J. Y. Gauthier , M. D. Percival , and D. Brömme , “Pharmacological Inhibition of Cathepsin S Decreases Atherosclerotic Lesions in Apoe‐/‐ mice,” Journal of Cardiovascular Pharmacology 56, no. 1 (2010): 98–105. 20410833 10.1097/FJC.0b013e3181e23e10

[228] H. Englert , J. Göbel , and D. Khong , “Targeting NETs Using Dual‐active DNase1 Variants,” Frontiers in immunology 14 (2023): 1181761. 37287977 10.3389/fimmu.2023.1181761 PMC10242134

[229] C. H. O. Meara , L. A. Coupland , and F. Kordbacheh , “Neutralizing the Pathological Effects of Extracellular Histones With Small Polyanions,” Nature Communications 11, no. 1 (2020): 6408. 10.1038/s41467-020-20231-y PMC7744542 33328478

[230] S. H. Chen , X. O. Scott , and Y. Ferrer Marcelo , “Netosis and Inflammasomes in Large Vessel Occlusion Thrombi,” Frontiers in Pharmacology 11 (2020): 607287. 33569001 10.3389/fphar.2020.607287 PMC7868597

[231] S. Yan , C. Fang , and L. Cao , “Protective Effect of Glycyrrhizic Acid on Cerebral Ischemia/Reperfusion Injury via Inhibiting HMGB1‐mediated TLR4/NF‐κB Pathway,” Biotechnology and Applied Biochemistry 66, no. 6 (2019): 1024–1030. 31545873 10.1002/bab.1825

[232] J. Zhu , H. Lu , and C. Guo , “Berberine Attenuates Ischemia‐reperfusion Injury Through Inhibiting HMGB1 Release and NF‐κB Nuclear Translocation,” Acta Pharmacologica Sinica 39, no. 11 (2018): 1706–1715. 30266998 10.1038/s41401-018-0160-1 PMC6289370

[233] E. Laridan , F. Denorme , and L. Desender , “Neutrophil Extracellular Traps in Ischemic Stroke Thrombi,” Annals of Neurology 82, no. 2 (2017): 223–232. 28696508 10.1002/ana.24993

[234] C. Winter , C. Silvestre‐Roig , and A. Ortega‐Gomez , “Chrono‐pharmacological Targeting of the CCL2‐CCR2 Axis Ameliorates Atherosclerosis,” Cell metabolism 28, no. 1 (2018): 175–182. 29861387 10.1016/j.cmet.2018.05.002

[235] M. Ferré‐Vallverdú , A. M. Latorre , and M. P. Fuset , “Neutrophil Extracellular Traps (NETs) in Patients With STEMI. Association With Percutaneous Coronary Intervention and Antithrombotic Treatments,” Thrombosis Research 213 (2022): 78–83. 35306431 10.1016/j.thromres.2022.03.002

[236] B. M. Everett , J. G. MacFadyen , T. Thuren , P. Libby , R. J. Glynn , and P. M. Ridker , “Inhibition of Interleukin‐1β and Reduction in Atherothrombotic Cardiovascular Events in the CANTOS Trial,” Journal of the American College of Cardiology 76, no. 14 (2020): 1660–1670. 33004131 10.1016/j.jacc.2020.08.011

[237] P. M. Ridker , B. M. Everett , and T. Thuren , “Antiinflammatory Therapy With Canakinumab for Atherosclerotic Disease,” New England Journal of Medicine 377, no. 12 (2017): 1119–1131. 28845751 10.1056/NEJMoa1707914

[238] K. Broch , A. K. Anstensrud , and S. Woxholt , “Randomized Trial of Interleukin‐6 Receptor Inhibition in Patients with Acute ST‐Segment Elevation Myocardial Infarction,” Journal of the American College of Cardiology 77, no. 15 (2021): 1845–1855. 33858620 10.1016/j.jacc.2021.02.049

[239] J. Tardif , S. Kouz , and D. D. Waters , “Efficacy and Safety of Low‐Dose Colchicine After Myocardial Infarction,” New England Journal of Medicine 381, no. 26 (2019): 2497–2505. 31733140 10.1056/NEJMoa1912388

[240] K. Vaidya , B. Tucker , and R. Kurup , “Colchicine Inhibits Neutrophil Extracellular Trap Formation in Patients with Acute Coronary Syndrome after Percutaneous Coronary Intervention,” Journal of the American Heart Association 10, no. 1 (2021): e018993. 33346683 10.1161/JAHA.120.018993 PMC7955504

[241] T. S. Opstal , A. van Broekhoven , and A. T. Fiolet , “Long‐Term Efficacy of Colchicine in Patients with Chronic Coronary Disease: Insights from LoDoCo2,” Circulation 145, no. 8 (2022): 626–628. 35188792 10.1161/CIRCULATIONAHA.121.058233

[242] S. M. Nidorf , A. T. Fiolet , and A. Mosterd , “Colchicine in Patients With Chronic Coronary Disease,” New England Journal of Medicine 383, no. 19 (2020): 1838–1847. 32865380 10.1056/NEJMoa2021372

[243] S. S. Jolly , M. d'Entremont , and S. F. Lee , “Colchicine in Acute Myocardial Infarction,” New England Journal of Medicine 392, no. 7 (2025): 633–642. 39555823 10.1056/NEJMoa2405922

[244] C. S. Lam , L. H. LUND , and S. J. SHAH , “Myeloperoxidase Inhibition in Heart Failure with Preserved or Mildly Reduced Ejection Fraction: SATELLITE Trial Results,” Journal of Cardiac Failure 30, no. 1 (2024): 104–110. 37072105 10.1016/j.cardfail.2023.04.003

[245] L. H. Lund , C. S. Lam , and P. E. Pizzato , “Rationale and Design of ENDEAVOR: A Sequential Phase 2b‐3 Randomized Clinical Trial to Evaluate the Effect of Myeloperoxidase Inhibition on Symptoms and Exercise Capacity in Heart Failure With Preserved or Mildly Reduced Ejection Fraction,” European Journal of Heart Failure 25, no. 9 (2023): 1696–1707. 37470101 10.1002/ejhf.2977 PMC10592288

[246] P. M. Ridker , M. Devalaraja , and F. M. M. Baeres , “IL‐6 Inhibition With ziltivekimab in Patients at High Atherosclerotic Risk (RESCUE): A Double‐blind, Randomised, Placebo‐controlled, Phase 2 Trial,” Lancet 397, no. 10289 (2021): 2060–2069. 34015342 10.1016/S0140-6736(21)00520-1

[247] P. M. Ridker , F. M. M. Baeres , and A. Hvelplund , “Rationale, Design, and Baseline Clinical Characteristics of the Ziltivekimab Cardiovascular Outcomes Trial: Interleukin‐6 Inhibition and Atherosclerotic Event Rate Reduction,” JAMA Cardiology 11, no. 1 (2026): 89–97. 41369941 10.1001/jamacardio.2025.4491

[248] J. W. Eikelboom , S. J. Connolly , and J. Bosch , “Rivaroxaban With or Without Aspirin in Stable Cardiovascular Disease,” New England Journal of Medicine 377, no. 14 (2017): 1319–1330. 28844192 10.1056/NEJMoa1709118

[249] S. Bozonet and A. Carr , “The Role of Physiological Vitamin C Concentrations on Key Functions of Neutrophils Isolated From Healthy Individuals,” Nutrients 11, no. 6 (2019): 1363. 31212992 10.3390/nu11061363 PMC6627200

[250] Y. Xu , H. Zheng , I. Slabu , E. A. Liehn , and M. Rusu , “Vitamin C in Cardiovascular Disease: From Molecular Mechanisms to Clinical Evidence and Therapeutic Applications,” Antioxidants (Basel) 14, no. 5 (2025): 506. 40427388 10.3390/antiox14050506 PMC12108419

[251] L. Bolognese and M. Felici , “Chronic Ischaemic Heart Disease and rivaroxaban: Which Patients Derive the Greatest Benefit?,” European Heart Journal 22, Suppl L (2020): L24–l27. 10.1093/eurheartj/suaa128 PMC7673606 33239976

[252] Y. Gong , B. Chen , Q. Tan , and W. Wei , “Neutrophil Extracellular Traps Enhance Procoagulant Activity and Predict Poor Prognosis in Patients with Metastatic Breast Cancer,” International Journal of General Medicine 18 (2025): 1247–1259. 40062357 10.2147/IJGM.S511024 PMC11890004

[253] K. Gollomp , M. Kim , and I. Johnston , “Neutrophil Accumulation and NET Release Contribute to Thrombosis in HIT,” JCI Insight 3, no. 18 (2018): e99445. 30232279 10.1172/jci.insight.99445 PMC6237233

[254] L. D. Healy , C. Puy , and A. Itakura , “Colocalization of Neutrophils, Extracellular DNA and Coagulation Factors During NETosis: Development and Utility of an Immunofluorescence‐based Microscopy Platform,” Journal of Immunological Methods 435 (2016): 77–84. 27286714 10.1016/j.jim.2016.06.002 PMC4935600

[255] A. Espiritu and K. M. O'Sullivan , “A Web of Challenges: The Therapeutic Struggle to Target NETs in Disease,” International Journal of Molecular Sciences 26, no. 10 (2025): 4773. 40429915 10.3390/ijms26104773 PMC12111817

[256] A. K. Meher , M. Spinosa , and J. P. Davis , “Novel Role of IL (Interleukin)‐1β in Neutrophil Extracellular Trap Formation and Abdominal Aortic Aneurysms,” Arteriosclerosis, Thrombosis, and Vascular Biology 38, no. 4 (2018): 843–853. 29472233 10.1161/ATVBAHA.117.309897 PMC5864548

[257] P. Libby and O. Soehnlein , “Inflammation in Atherosclerosis: Lessons and Therapeutic Implications,” Immunity 58, no. 10 (2025): 2383–2401. 41045921 10.1016/j.immuni.2025.09.012

[258] N. Potere , A. Bonaventura , and A. Abbate , “Novel Therapeutics and Upcoming Clinical Trials Targeting Inflammation in Cardiovascular Diseases,” Arteriosclerosis, Thrombosis, and Vascular Biology 44, no. 12 (2024): 2371–2395. 39387118 10.1161/ATVBAHA.124.319980 PMC11602387

[259] N. Bouabdallaoui , J. Tardif , and D. D. Waters , “Time‐to‐treatment Initiation of Colchicine and Cardiovascular Outcomes After Myocardial Infarction in the Colchicine Cardiovascular Outcomes Trial (COLCOT),” European Heart Journal 41, no. 42 (2020): 4092–4099. 32860034 10.1093/eurheartj/ehaa659 PMC7700755

[260] C. Laudani , A. Abbate , D. J. Angiolillo , and M. Galli , “CLEAR Results, Cloudy Impact: Colchicine's Neutral Role in ST‐segment Elevation Myocardial Infarction,” European Heart Journal ‐ Cardiovascular Pharmacotherapy 11, no. 3 (2025): 215–217. 40080070 10.1093/ehjcvp/pvaf011 PMC12046578

[261] Y. Li , S. Chen , and Y. Yang , “Colchicine Inhibits NETs and Alleviates Cardiac Remodeling After Acute Myocardial Infarction,” Cardiovascular Drugs and Therapy 38, no. 1 (2024): 31–41. 35900652 10.1007/s10557-022-07326-y

[262] C. Schauer , C. Janko , and L. E. Munoz , “Aggregated Neutrophil Extracellular Traps Limit Inflammation by Degrading Cytokines and Chemokines,” Nature Medicine 20, no. 5 (2014): 511–517. 10.1038/nm.3547 24784231

[263] S. L. Wong , M. Demers , and K. Martinod , “Diabetes Primes Neutrophils to Undergo NETosis, Which Impairs Wound Healing,” Nature Medicine 21, no. 7 (2015): 815–819. 10.1038/nm.3887 PMC4631120 26076037

[264] E. A. Middleton , X. He , and F. Denorme , “Neutrophil Extracellular Traps Contribute to Immunothrombosis in COVID‐19 Acute respiratory Distress Syndrome,” Blood 136, no. 10 (2020): 1169–1179. 32597954 10.1182/blood.2020007008 PMC7472714

[265] A. Hidalgo , P. Libby , O. Soehnlein , I. V. Aramburu , V. Papayannopoulos , and C. Silvestre‐Roig , “Neutrophil Extracellular Traps: From Physiology to Pathology,” Cardiovascular Research 118, no. 13 (2022): 2737–2753. 34648022 10.1093/cvr/cvab329 PMC9586562

[266] P. Li , M. Li , M. R. Lindberg , M. J. Kennett , N. Xiong , and Y. Wang , “PAD4 is Essential for Antibacterial Innate Immunity Mediated by Neutrophil Extracellular Traps,” Journal of Experimental Medicine 207, no. 9 (2010): 1853–1862. 20733033 10.1084/jem.20100239 PMC2931169

[267] E. Kolaczkowska , C. N. Jenne , and B. G. J. Surewaard , “Molecular Mechanisms of NET Formation and Degradation Revealed by Intravital Imaging in the Liver Vasculature,” Nature Communications 6 (2015): 6673. 10.1038/ncomms7673 PMC4389265 25809117

[268] A. Zernecke , H. Winkels , and C. Cochain , “Meta‐Analysis of Leukocyte Diversity in Atherosclerotic Mouse Aortas,” Circulation Research 127 (2020): 402–426. 32673538 10.1161/CIRCRESAHA.120.316903 PMC7371244

[269] M. T. Bertran , R. Walmsley , and T. Cummings , “A Cyclic Peptide Toolkit Reveals Mechanistic Principles of Peptidylarginine Deiminase IV Regulation,” Nature Communications 15, no. 1 (2024): 9746. 10.1038/s41467-024-53554-1 PMC11555231 39528459

[270] X. Geng , D. Wang , and H. Li , “The Pivotal Role of Neutrophil Extracellular Traps in Cardiovascular Diseases: Mechanisms and Therapeutic Implications,” Biomedicine & Pharmacotherapy 179 (2024): 117289. 39151311 10.1016/j.biopha.2024.117289

[271] T. Kofler , R. Kurmann , and D. Lehnick , “Colchicine in Patients with Coronary Artery Disease: A Systematic Review and Meta‐Analysis of Randomized Trials,” Journal of the American Heart Association 10, no. 16 (2021): e021198. 34369166 10.1161/JAHA.121.021198 PMC8475038

[272] G. Huang , H. Wu , and B. Lin , “Targeting Neutrophil Extracellular Traps: A New Strategy for the Treatment of Acute Ischemic Stroke Based on Thrombolysis Resistance,” Seminars in Thrombosis and Hemostasis 52, no. 1 (2026): 80–91. 40461018 10.1055/a-2609-3457 PMC12799313

[273] G. A. Mensah , V. Fuster , C. J. Murray , et al., “Global Burden of Cardiovascular Diseases and Risks, 1990–2022,” Journal of the American College of Cardiology 82, no. 25 (2023): 2350–2473. 38092509 10.1016/j.jacc.2023.11.007 PMC7615984

